# Machine‐learning prediction of affinity and epistasis in the bovine pancreatic trypsin inhibitor–chymotrypsin complex

**DOI:** 10.1002/pro.70660

**Published:** 2026-06-05

**Authors:** Noam Tzuri, Itamar Kass, Yaron Orenstein, Niv Papo

**Affiliations:** ^1^ Avram and Stella Goldstein‐Goren Department of Biotechnology Engineering Ben‐Gurion University of the Negev Beer‐Sheva Israel; ^2^ National Institute of Biotechnology Ben‐Gurion University of the Negev Beer‐Sheva Israel; ^3^ The Ilse Katz Institute for Nanoscale Science and Technology Ben‐Gurion University of the Negev Beer‐Sheva Israel; ^4^ Department of Computer Science Bar‐Ilan University Ramat Gan Israel; ^5^ The Mina and Everard Goodman Faculty of Life Sciences Bar‐Ilan University Ramat Gan Israel

**Keywords:** bovine pancreatic trypsin inhibitor, chymotrypsin, deep mutational scanning, machine learning, neural networks, protein engineering, protein–protein interactions

## Abstract

Protein–protein interactions (PPIs) are shaped by evolutionary pressures that fine‐tune binding affinities and drive the epistatic relationships that support functional outcomes. Here, we used the complex of bovine pancreatic trypsin inhibitor (BPTI) and chymotrypsin as a model system to study how mutations at one or two positions affect binding affinity and epistasis. To predict the binding affinity landscape of the BPTI–chymotrypsin complex, we combined deep sequencing data, obtained from a saturation scanning mutagenesis BPTI library, with a machine‐learning (ML) model. Using this ML model, which was trained on a subset of experimental binding data, we predicted the binding affinities and epistatic interactions across thousands of single and double BPTI mutants, including those not observed in the library. Our predictive approach completed missing data points and enabled us to reveal global trends in affinity changes and mutation couplings within specific binding interface positions. Our analysis revealed that different mutations in the same position may have different effects on affinity, with most double mutations leading to increased epistasis, particularly at hotspot positions, thereby indicating a cooperative binding effect. In most cases, affinity and epistasis were inversely correlated, with affinity enhancement of double‐mutant variants being associated with negative epistasis. Our approach can be readily generalized to predict mutation effects in larger combinatorial libraries and in proteins for which structural information is lacking.

## 1 INTRODUCTION

Protein–protein interactions (PPIs) are central to many biological functions—from signaling to immune recognition. These interactions, which are shaped by natural evolution, depend on specific residues at the binding interface, where mutations (even a few) can significantly strengthen or weaken affinity (Kortemme & Baker, 2002). While some positions are critical for binding (i.e., hotspots) (Lin et al., 2012), others offer opportunities for improvements in binding affinity (i.e., coldspots) (Shirian et al., 2016).

Unlike natural evolution, which balances multiple constraints, such as affinity, specificity, solubility, and/or stability, library‐assisted directed protein screening allows us to focus on optimizing a single function—in this case affinity. Although mutations at single positions may have a strong effect on affinity, they are often influenced by residues at other positions, a phenomenon known as epistasis (Lipsh‐Sokolik & Fleishman, 2024). This interdependence between residues adds complexity to the process of protein design and highlights the importance of mapping mutational effects in a combinatorial context in a large sequence space. A method widely used to enable a broader exploration of protein sequence space comprises screening large libraries of protein variants. Although this method for mapping the binding landscapes of protein complexes provides a robust platform that is both scalable and effective, library screening suffers from two major drawbacks, namely, multiple rounds are required to isolate optimal variants and the resolution of the full mutational landscape remains limited.

More recently, high‐throughput methods, such as deep mutational scanning (DMS) coupled with high‐throughput sequencing (HTS), have enabled the screening, in a single experiment, of large libraries encompassing thousands of variants (Fowler et al., 2014; Fowler & Fields, 2014). These experimental approaches may be integrated with computational analyses to interpret large datasets, to identify key determinants of function, and to guide further study design. Recent work has demonstrated that DMS can be used not only to map affinity or selectivity landscapes (Aharon et al., 2020; Heyne et al., 2021; Naftaly et al., 2018) but also to systematically quantify epistasis (Judge et al., 2024), thereby uncovering extensive networks of cooperative and compensatory interactions between residues within protein binding interfaces. Such an approach could similarly be applied to various protein complexes to guide library‐assisted protein design. However, even the above‐mentioned advanced techniques have the inherent limitation that the combinatorial explosion of the sequence space as the number of mutated positions increases makes it impossible to generate and test all possible variants.

To address the limitations of incomplete experimental coverage of protein libraries, machine‐learning (ML) approaches have been increasingly applied to guide protein design. For example, ML‐guided directed evolution has been applied to predict mutation effects and to prioritize promising variants, thereby greatly improving the efficiency of protein engineering and optimization (Guo & Yamaguchi, 2022; Wu et al., 2019; Yang et al., 2019). Notably, ML, particularly deep neural networks, has been successfully used to predict protein function, as exemplified in a number of ways—by the implementation of the ProBASS model to predict the effect of mutations on binding affinity (Gurusinghe et al., 2024); by the application of deep learning to model the affinity landscapes of PPIs (Meiri et al., 2024); and by the power of neural networks in protein sequence design (Dauparas et al., 2022). These advances establish ML as a key tool for overcoming experimental limitations and guiding rational protein design.

In the current study, we leveraged ML to study the PPI between the serine protease inhibitor, bovine pancreatic trypsin inhibitor (BPTI), and chymotrypsin and—through that interaction—the affinity landscape of BPTI, a well‐characterized 58‐amino‐acid globular protein that tolerates multiple mutations without disruptions in its fold or its activity. The broad specificity and high stability of BPTI make it an ideal model for studying protein conformation and the molecular basis of PPIs (Ascenzi et al., 2003). Importantly, its binding to chymotrypsin is mediated exclusively by direct amino acid interactions, independent of any post‐translational modifications. Chymotrypsin, which binds BPTI with moderate affinity (*K*_*i*_ = 10^−8^ M) (Castro & Anderson, 1996), serves as a suitable protease partner for exploring BPTI's affinity landscape. ML models have thus been applied to study the BPTI–chymotrypsin complex (Gurusinghe et al., 2024); for example, in a study investigating affinity and epistasis of the BPTI–chymotrypsin complex, Heyne et al. generated a library of single and double mutants limited to 12 interfacial positions in BPTI (Heyne et al., 2021). However, in that study, only approximately half of the possible double‐mutant variants were present in the library, limiting the completeness of the binding landscape.

In the current study, we developed an ML‐based method to predict the binding affinities of all possible double‐mutant BPTI variants, including those not included in the above‐described library, thereby enabling the construction of the full binding landscape of a protein with two mutations. Using a supervised learning approach, we trained our model to predict the binding affinity (ΔΔ*G*_bind_) of single and double BPTI mutants to chymotrypsin, and on the basis of those predictions, we were then able to predict the epistatic effect of double mutations in all double‐mutant variants. The predictions revealed trends in affinity and epistasis that deepened our understanding of the BPTI–chymotrypsin interaction landscape. We were also able to dissect out the roles played by the characteristics (e.g., charge, hydrophobicity) of each amino acid in affinity and epistasis. In general, an ML‐based method such as ours could be expanded to predict libraries with mutations at more than the 12 interface positions tested in the current study as well as to cover sequences whose structures are currently unknown.

## 2 RESULTS

**FIGURE 1:**
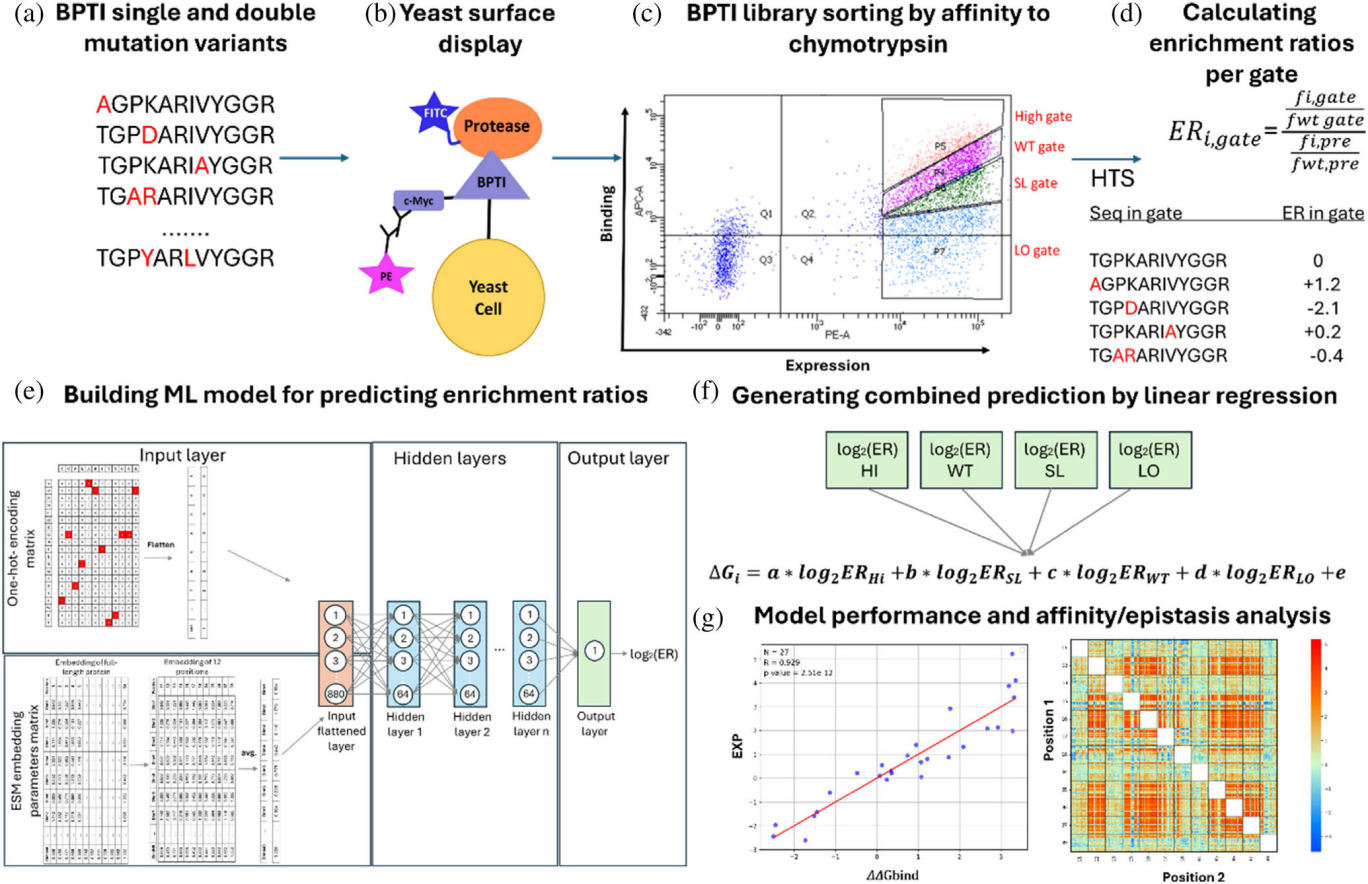
Overview of our experimental and computational workflow for identifying the affinity/epistasis landscape of bovine pancreatic trypsin inhibitor (BPTI) in complex with chymotrypsin. (a) A BPTI‐derived library containing single‐ and double‐mutant variants was generated (Heyne et al., 2021). (b) and (c) The library was yeast surface displayed, subjected to flow cytometry screening for affinity to chymotrypsin, and sorted using four gates, each having different affinities to chymotrypsin. The fractions were then analyzed by HTS (Heyne et al., 2021). (d) and (e) This study: Computational analysis of the HTS results (Heyne et al., 2021) was followed by calculation of the frequency of each variant in each of the four gates, thereby generating a sequence–log_2_ ER dataset, where ER—enrichment ratio. (E) Based on the log_2_ ER values, a neural network was trained to accurately and quantitatively predict the impact of unobserved mutations on the binding affinity. (F) log_2_ ER values from all gates were combined into a single ΔΔ*G*_bind_ prediction by using linear regression. (G) The final step comprised evaluation of the model performance and analysis of the affinity/epistasis landscape.

An overview of our experimental and computational workflow is presented in Figure 1.

### 2.1 Re‐processing BPTI–chymotrypsin deep mutational scanning data

To predict the affinity and epistasis of double‐mutant BPTI variants in a complex with chymotrypsin, we reanalyzed the HTS data previously produced by Heyne et al. (2021). That data was based on a BPTI library of variants having one to two mutations at 12 different positions, namely, positions 11–13, 15–18, 34–37, or 39. Enrichment of the library, via a yeast‐surface‐display‐based screening method, enabled the sorting of the BPTI variants into four gates (fractions) according to their affinity to chymotrypsin; those gates were designated high affinity (HI), wild‐type (WT)‐like affinity, slightly lower (SL) affinity than wild type, and low affinity (LO). In the current study, for each gate we filtered out sequences of variants that lacked the TAGC primer (the start of the protein‐coding region) and those that were too short, contained invalid nucleotide characters (i.e., not A, C, G, or T), or had mutations in positions other than the 12 pre‐defined positions (at the amino‐acid level); the BPTI sequence of amino acids 1–54 was used as a reference. We successfully recovered a total of 258033, 254909, 279096, and 214380 amino‐acid sequences from the HI, WT, SL, LO gates, respectively, and 474383 amino‐acid sequences from the pre‐sort library. We then calculated the log_2_ ER (Equations ((1), (3))–((1), (3))) of each variant, which is the relative enrichment of that variant in a particular gate compared to the pre‐sort library.

### 2.2 Training our models to predict the affinity of BPTI variants

Out of the 228 potential single‐mutant BPTI variants in the library (19 amino‐acid substitutions × 12 positions), we observed 213 (~93.4%), 227 (~99.6%), 228 (100%), 228 (100%), and 228 (100%) variants in the HI, WT, SL, and LO gates and the pre‐sort library, respectively. Out of the 23,826 potential double‐mutant BPTI variants (19 × 19 double amino‐acid substitutions × 66 pairs of positions), we observed only 3383 (~14.2%), 4943 (~20.7%), 12,853 (~53.9%), 15,768 (~66.2%), and 21,179 (~88.9%) in the HI, WT, SL, and LO gates and the pre‐sort library, respectively. To impute the missing space of the double‐mutant variants, we trained an ML model over each gate to predict the log_2_ ER value of any given variant. To train the four models, we used the dataset of observed single‐ and double‐mutant variants and their respective log_2_ ER values as labels.

#### 2.2.1 Hyperparameter search and model performance evaluation

For each of the four ML models, we held out 20% of high‐quality variants (i.e., the most frequent variants according to their total read count in the pre‐sort library and the selection gates). Of these 20%, we used half for testing the trained model (the most frequent variants in the held‐out set) and the other half for hyperparameter and model search, which included neural‐network architecture and input representation selections. We trained each model on the remaining 80% of the variants. For the hyperparameter and model optimization, we assessed the performance of the model for each gate by using the Pearson correlation of the predicted log_2_ ER values with the measured log_2_ ER values on the validation set (Table 1). The rationale for using the Pearson correlation was that it enabled us to rank the variants by relative enrichment across the gates.

**TABLE 1:** Hyperparameter search range for fully connected NN and optimal values.

Hyperparameter	Search range	HI gate	WT gate	SL gate	LO gate
Batch size	{32, 64, 128, 256, 512}	256	32	32	32
Number of epochs	{10, 20, 30, 40, 50}	50	30	20	30
Optimizer learning rate	{10^−5^, 10^−4^, 5 × 10^−4^, 10^−3^}	10^−3^	10^−3^	10^−3^	10^−3^
Layers in the architecture	{3, 4, 5}	[64, 32, 32, 32]	[64, 32, 32]	[64, 64, 32, 32]	[64, 64, 32, 32, 32]
Neurons in the architecture's layers	{32, 64, 128, 256}				
Dropout	{0,0.1, 0.2, 0.3, 0.4, 0.5}	0.1	0.3	0.1	0.2
Pearson correlation (validation set)	‐	0.871	0.830	0.840	0.763
Pearson correlation (test set)	‐	0.861	0.650	0.750	0.803

**FIGURE 2:**
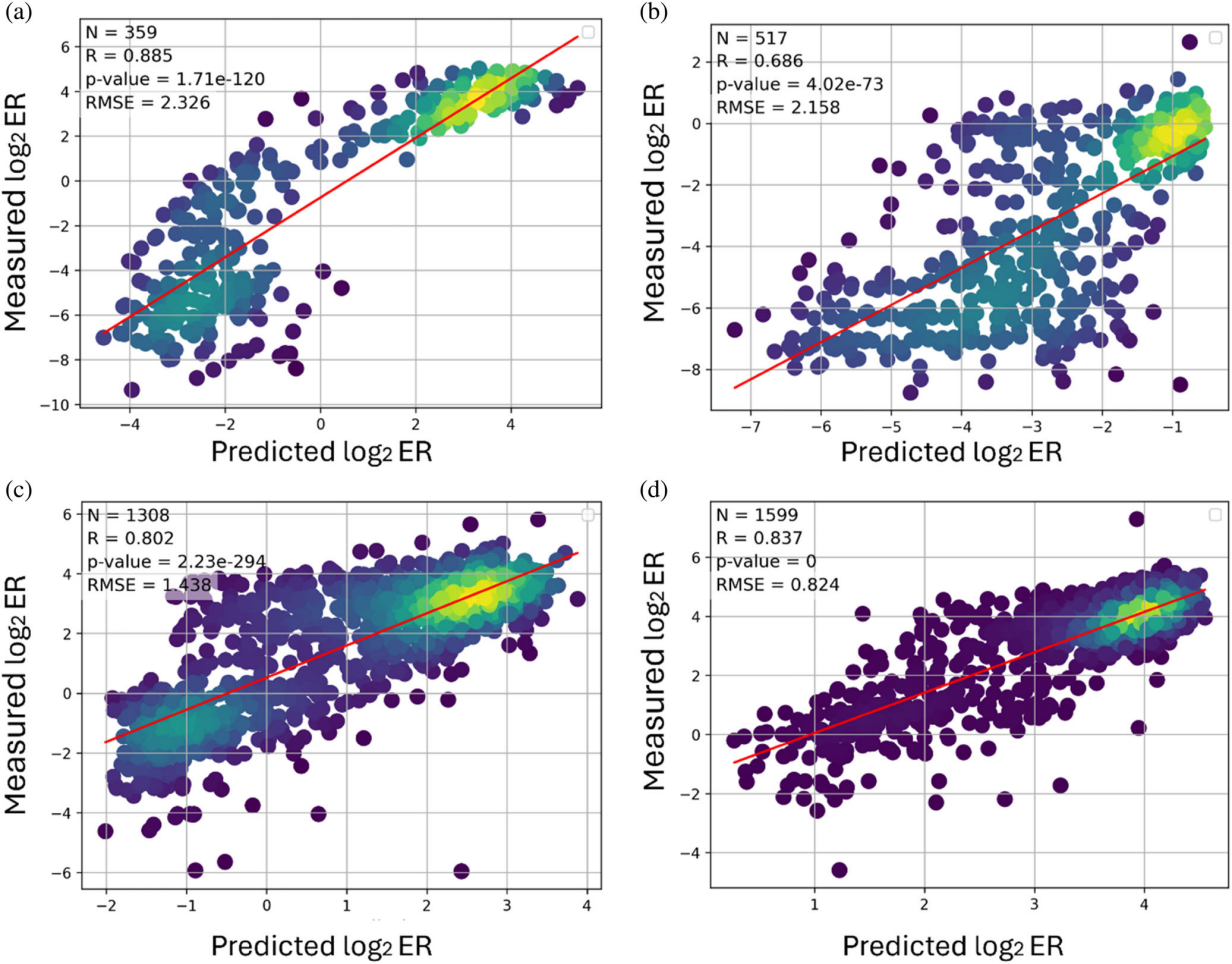
Predictive performance of our four ML models. We trained each model on 90% of the data and evaluated it on the top 10% of the most frequent variants of the (a) HI, (b) WT, (c) SL, and (d) LO gates. N—number of datapoints in the test set; *R*—Pearson correlation; RMSE—root mean square error.

Among the evaluated architectures and input representations, the best performance was obtained for two model configurations, where one received only ESM2‐derived features (*R* = 0.827) while the other received both one‐hot and ESM2‐derived features (*R* = 0.826), which we selected for its hybrid input representation (Table S1). After hyperparameter and model architecture and input representation search, we re‐trained the models on 90% of the variants and evaluated each on its respective held‐out 10% test set. Pearson correlations of 0.885, 0.686, 0.802, and 0.837 were obtained for the HI, WT, SL, and LO gates, respectively (Figure 2a–d). An additional evaluation of the model for each gate in a random train‐test split confirmed the high performance of the models, with Pearson correlations of 0.869, 0.749, 0.756, and 0.618 for the HI, WT, SL, and LO gates, respectively (Figure S1). The high correlations thus highlight the ability of our models to accurately predict log_2_ ER values.

#### 2.2.2 Evaluating the prediction of absolute binding affinities

**FIGURE 3:**
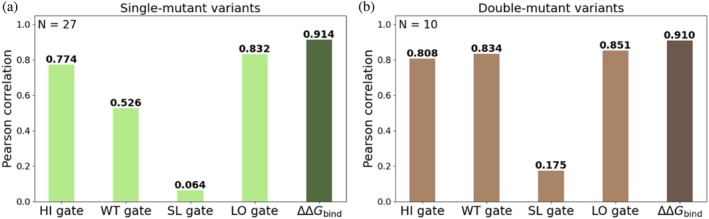
Pearson correlations between predicted log_2_ ERs and experimental absolute binding affinities for each gate individually (light shades) and for the combined ΔΔ*G*_bind_ values (dark shades) for (a) 27 single‐mutant variants and (b) 10 double‐mutant variants.

We tested the ability of our four models to predict experimental binding data for purified proteins. We curated ΔΔ*G*_bind_ chymotrypsin‐binding measurements of 27 BPTI single‐mutant variants from the literature (Castro & Anderson, 1996; Krowarsch et al., 1999). In this evaluation, we removed these 27 variants from the training sets. The absolute Pearson correlations between the predicted ERs and the experimental ΔΔ*G*_bind_ values were 0.774, 0.526, 0.064, and 0.832 for the HI, WT, SL, and LO models, respectively (Figures 3a and S2). The highest correlations with the experimental measurements of the purified proteins were obtained for the HI and LO models.

We also tested the ability of our models to predict the binding affinities of double‐mutant variants. We curated affinity (ΔΔ*G*_bind_) values from previous studies of 10 double‐mutant BPTI variants (Buczek et al., 2002; Grzesiak et al., 2000; Kiczak et al., 2001) and used those values as a held‐out test set. Similar to the evaluation for the single mutants, we excluded these 10 variants from the training set of each model. The absolute Pearson correlations between predicted log_2_ ER values and experimental ΔΔ*G*_bind_ values were 0.808, 0.834, 0.175, and 0.851 for the HI, WT, SL, and LO models, respectively (Figures 3b and S3). The HI, WT, and LO models performed the best, with particularly high agreement being observed for the HI and LO gates. We thus concluded that the HI and LO gates may be particularly informative for predicting the binding affinities of double mutants.

### 2.3 Integrating affinity‐gate predictions to estimate ΔΔ*G*_bind_

#### 2.3.1 Improving ΔΔ*G*_bind_ prediction by combining all affinity gates

To predict the absolute binding affinity of each variant, we developed a linear regression model that converts the log_2_ ER values derived from ML predictions into predicted ΔΔ*G*_bind_ values. We fitted a linear regression model on the 27 experimentally measured ΔΔ*G*_bind_ values by combining the log_2_ ER values of the four gates (ER_HI_, ER_SL_, ER_WT_, ER_LO_) (Equation (4)). The resulting model showed an excellent fit to the training data (*R* = 0.914; Figures 3a and S4A). While the HI and LO gate models showed relatively high correlations (*R* = 0.774 and 0.832, respectively), the combined ΔΔ*G*_bind_ prediction achieved an even higher correlation (Figure 3a). We posit that this improvement arose because the combination prediction served to neutralize the gate‐specific noise or biases introduced by each affinity gate. Integrating information from all four gates likely reduces these errors, leading to more accurate and robust predictions.

We then applied this approach to predict ΔΔ*G*_bind_ of double‐mutant variants and used the same equation that we generated to fit the 27 single mutants to predict the ΔΔ*G*_bind_ of the double mutants. This approach yielded a high correlation (*R* = 0.910; Figures 3b and S4B), exceeding the correlations for the individual gates (*R* = 0.808, 0.834, and 0.851 for the HI, WT and LO gates, respectively; Figure 3b).

#### 2.3.2 Comparing double‐mutant ΔΔ*G*_bind_ predictions with an additive baseline

To directly compare the combined ΔΔ*G*_bind_ predictions with a simple additive baseline, we calculated an additive prediction for each double mutant as the sum of the predicted ΔΔ*G*_bind_ values of the two corresponding single mutants. We then compared both the direct double‐mutant predictions and the additive predictions against the experimental ΔΔ*G*_bind_ values for the same 10 double mutants, which we excluded with their single‐mutant counterparts from the training set in this analysis. Since this test set included only 10 double‐mutant variants, we performed bootstrapping (i.e., report the mean and standard deviation over all 10‐, 9‐ and 8‐subsets) to enhance robustness. The direct predictions of double‐mutant variants achieved a Pearson correlation with the experimental values greater by several standard deviations compared to the additive baseline (*R* = 0.930 ± 0.014 and *R* = 0.874 ± 0.040, respectively). These results indicate that the direct model modestly improves the ranking of double‐mutant affinities relative to an additive prediction.

### 2.4 Binding affinity landscape of the BPTI–chymotrypsin complex

#### 2.4.1 Complete binding affinity landscape

**FIGURE 4:**
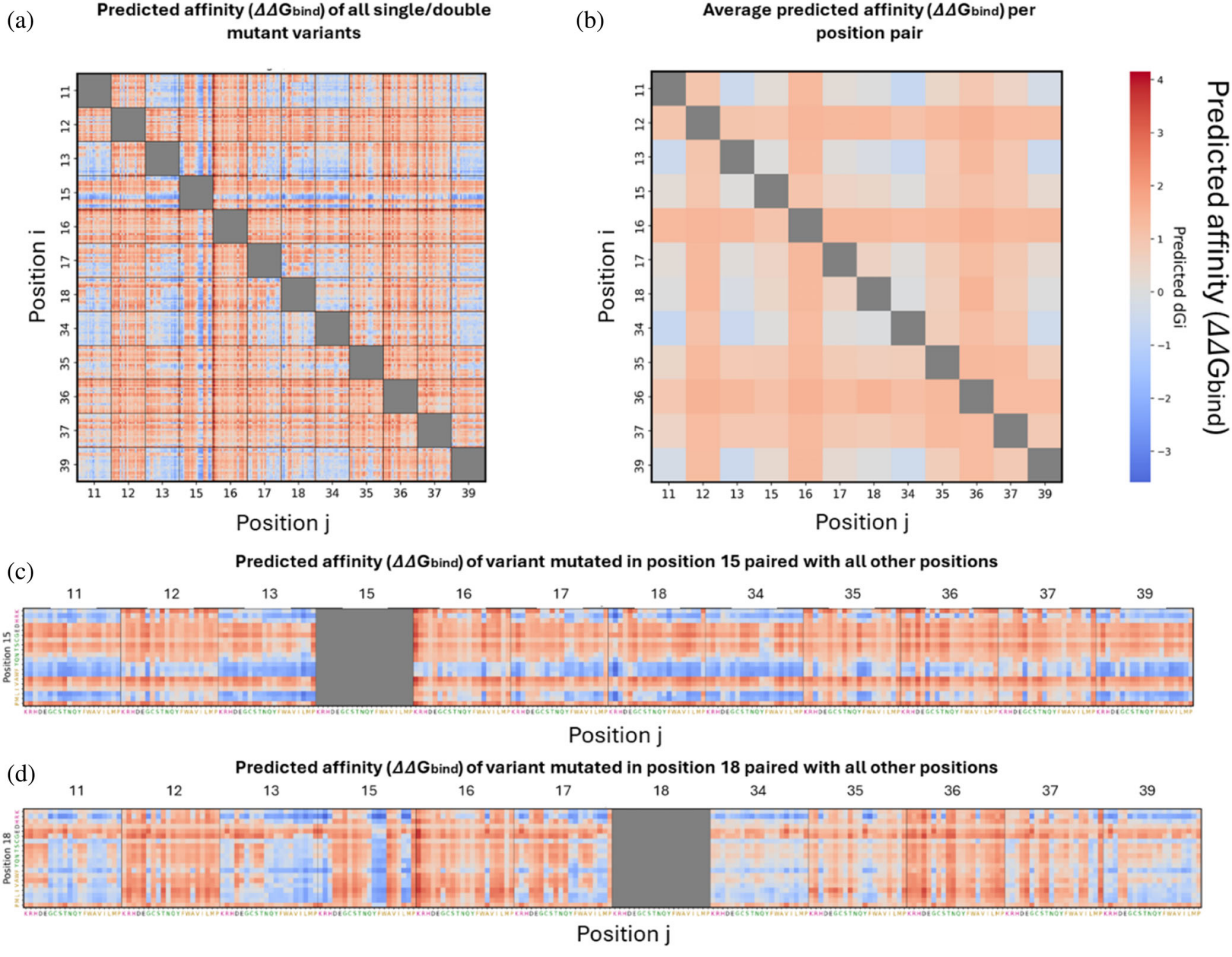
Affinity landscape of single‐ and double‐mutant BPTI variants in the BPTI–chymotrypsin complex. (a) Affinity (ΔΔ*G*_bind_) values for all variants. (b) Average ΔΔ*G*_bind_ for each pair of mutated positions. (c) and (d) ΔΔ*G*_bind_ for each variant mutated at (c) position 15 or (d) position 18 with all other 11 BPTI positions.

We generated the binding affinity landscapes of BPTI–chymotrypsin complexes for all single‐ and double‐mutant variants (Figure 4a). We then computed the average affinity (ΔΔ*G*_bind_) obtained from mutating two positions simultaneously (Figure 4b), thereby enabling us to assess the influence of individual positions as well as the combined effect of pairs of positions on the affinity between BPTI and chymotrypsin. We observed that mutations at positions 12, 16, and 36, and to a lesser extent at positions 35 and 37, led to a decrease in the average ΔΔ*G*_bind_ when combined with mutations at other positions, suggesting that the above five positions can be identified as hotspots (Figure 4b). Heyne et al. similarly identified positions 12, 16, 36, and 37 as hotspots on the basis of an analysis of their experimental data (Heyne et al., 2021). In contrast, when positions 11, 13, and 34 were mutated simultaneously, we observed an improvement in affinity, indicating a cooperative effect between these positions. Notably, positions 11, 13, 34, and 39 clustered spatially within the structure, suggesting cooperative local interactions, whereas adjacent positions 12 and 16 acted as hotspots, likely reflecting their essential role in maintaining the binding interface. Examination of a higher‐resolution heatmap of the affinity values for all single and double mutants revealed that, for some positions, amino acids with similar physicochemical properties (such as positively charged, negatively charged, hydrophobic, or polar residues) tended to have similar effects on affinity (Figure 4a). For example, when Lys 15 in BPTI was mutated to another positively charged or aromatic residue or to Leu or Met, the affinity of BPTI for chymotrypsin was enhanced (Figure 4c). At the same position, negatively charged, hydrophilic residues led to a decrease in affinity, as did certain hydrophobic residues, such as Val, Ala, Ile, and Pro. These findings are in agreement with a previous study that determined the affinities of multiple variants mutated at position 15 (Krowarsch et al., 1999).

To study the influence of the physicochemical properties of the amino acid residues in BPTI on its affinity to chymotrypsin at the atom level, we used the physics‐based FoldX Suite to model different mutations at position K15. Our analysis showed that the mutations K15R and K15H increased the affinity of BPTI toward chymotrypsin, with the enhanced affinity being due mainly to the hydrogen bonds formed between the side chain of the residue at position 15 and the surrounding residues. Notably, at position 15, Lys has three hydrogen bonds and Arg has four, but His has only one. In contrast, our FoldX‐based calculations suggested that the reduction in hydrogen bonds for His was accompanied by an increase in the sidechains' entropy and hence that the overall binding energy of BPTI‐K15H was favorable for binding. The physics‐based model indicated that the sidechains of D15 and E15 did not form hydrogen bonds with their surrounding residues, resulting in less favorable conditions for the formation of BPTI‐chymotrypsin complexes. Our calculations for a different set of mutations involving small‐to‐medium amino acids, such as K15V, K15A, K15I, and K15P, showed that the binding energy (compared to that for the above four mutations at K15) was positive, again meaning that the conditions for complex formation were unfavorable. This was mainly because a hydrophobic residue (in BPTI) being buried within a region (in chymotrypsin) with many polar groups. An exception to this explanation is demonstrated for K15P, in which a large determinant is the BPTI backbone strain due to the rigidity of Pro. This strain likely introduces backbone displacement, which has a negative effect on the binding affinity (Figure S5). Yet another mutation at position 15, namely K15L, which results in different physicochemical properties, was also experimentally found to increase the BPTI‐chymotrypsin interactions. Our modeling results are consistent with these experimental findings. Our calculations show that this affinity enhancement of K15L is due mainly to the lack of van‐der‐Waals clashes between the L15 sidechain and the surrounding residues (in chymotrypsin's S1 pocket) and the absence of steric hindrance in comparison to I15 and V15, which both have *β*‐branched sidechains. The binding preference for L15 over I15 and V15 is consistent with other structural analyses of BPTI‐chymotrypsin complexes (Helland et al., 2003).

Another example is provided by the substitution of Ile18: Upon substitution of Ile with a positively charged residue, affinity was improved (except when position 18 was combined with hotspot positions as partner positions), whereas substitution of Ile at the same position with a negatively charged residue or with Pro or Gly resulted in decreased affinity (Figure 4d).

#### 2.4.2 Binding affinity per position

**FIGURE 5:**
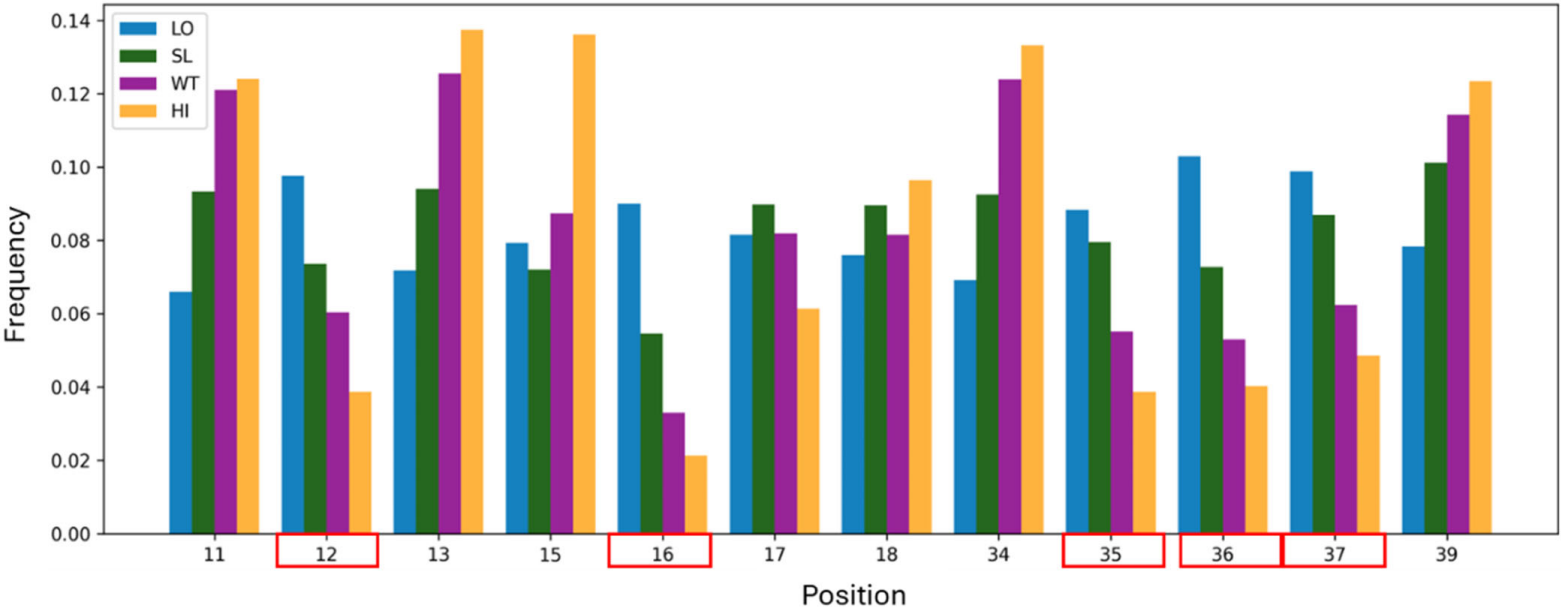
Frequency of variants mutated per position in each gate. The positions indicated in red boxes were identified as hotspot positions.

To characterize positional effects, we analyzed the distribution of mutations in the four affinity gates. For each of the 12 relevant positions, we calculated the frequency of variants falling in each gate. There was a higher representation of variants mutated at positions 12, 16, and 35–37 in the LO gate than in the HI gate (Figure 5). This enrichment supports the identification of these five positions as affinity hotspots, as mutations at these positions are strongly associated with reduced binding affinity. The fact that substitutions at these positions frequently resulted in low‐affinity variants reinforces the conclusion that these residues (i.e., 12, 16 and 35–37) play critical structural and functional roles in maintaining the binding interface.

To further evaluate the ability of our models to predict changes in affinity that result from changes for all residues in BPTI (including positions “unseen” by the model), we performed a position‐wise holdout evaluation. For each iteration, all variants (single and double mutants) containing a mutation at a specific position were excluded from the training set and used exclusively as the test set. Thus, the model had to predict the effect of mutations at a specific position based solely on information learned from other positions. The predictive performance of the models varied substantially between positions, with positions 34, 39, and 13 exhibiting the highest average correlations across the gates (average correlations 0.654, 0.628, and 0.602, respectively), indicating that their mutational effects were governed largely by generalizable sequence patterns. In contrast, positions 16, 12, and 15 gave low performance (average correlations 0.175, 0.341, and 0.349, respectively) (Table S2). Notably, positions previously identified as affinity hotspots were among the most difficult to predict under this strict positional holdout scenario. Although low‐affinity variants were enriched with hotspots, their effects did not generalize to unseen positions, suggesting that their contribution to binding is highly position specific.

To further assess the implications of position‐specific information, we repeated the analysis using a subset of the position‐wise held‐out variants. For each target position, 10% of variants from the original held‐out set were reserved as an independent test set. Two models were then trained—one excluding all target‐position variants, as in the strict position‐wise holdout, and one including the remaining target‐position variants not used for testing. Across all four affinity gates, the latter model consistently gave a higher correlation between predicted and measured log_2_ ER values, indicating that access to target‐position variants improves model performance (Figure S6).

### 2.5 Epistasis prediction and landscape analysis

#### 2.5.1 Epistasis landscape of BPTI–chymotrypsin complexes

**FIGURE 6:**
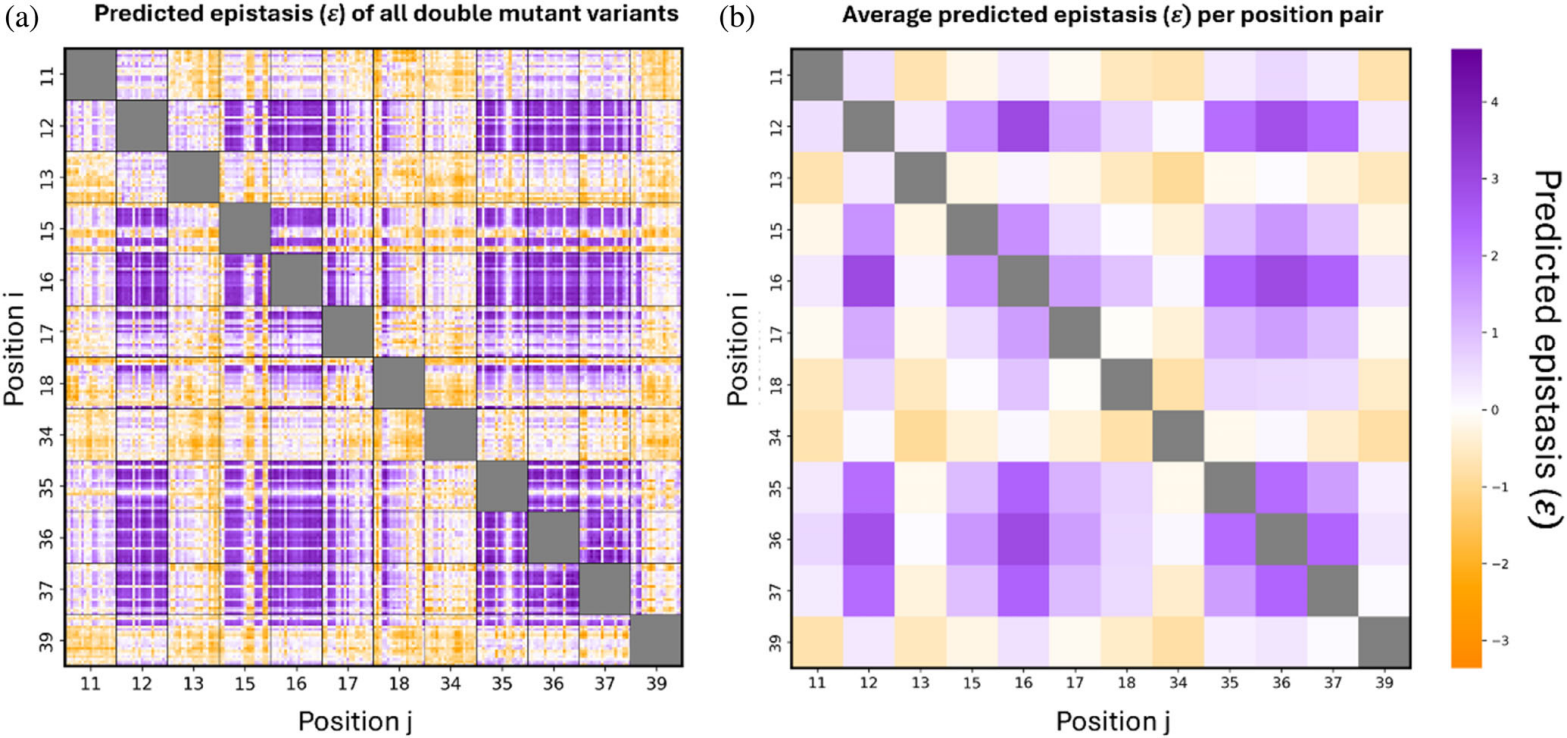
Epistasis landscape of double‐mutant variants. (a) Epistasis (𝜀) values for each variant. (b) Average 𝜀 for each pair of mutated positions.

To evaluate the impact of two mutated positions on binding affinity compared to the sum of individual changes, we predicted the epistasis (denoted as 𝜀) of all double mutants. We identified double mutations with relatively high positive or negative epistasis by calculating the difference between the binding affinity value of a double mutant (ΔΔ*G*_bind,ij_) versus the sum of binding affinity values of its single mutants (ΔΔ*G*_bind,*i*_ and ΔΔ*G*_bind,*j*_) (Equation (5)). Theoretically, negative (𝜀< 0) or positive (𝜀 >0) epistasis is obtained for double mutants when the affinity is lower or higher, respectively, than that expected from the independent contributions to affinity (i.e., the additive effect) of each of the two single mutations. Ideally, mutations in two positions are defined as additive if the mutations have neither positive nor negative epistasis effects, that is, 𝜀 = 0, but in practice affinity may be regarded as additive when 𝜀 is relatively close to 0. We thus predicted 𝜀 for each double mutation (Equation (5)) (Figure 6a). We also averaged 𝜀 for each pair of mutated positions (Figure 6b), thereby enabling us to determine the role played by specific positions in determining epistasis.

The predicted epistasis landscape of all double mutants revealed both positive and negative patterns (Figure 6a). Averaging epistasis across all amino‐acid substitutions for each pair emphasized consistent positional trends (Figure 6b). Notably, pairing hotspot positions often resulted in positive average epistasis, indicating cooperative effects when residues in these key positions were simultaneously mutated. In contrast, the pairing of positions 13 and 34 with many other positions gave predominantly negative average epistasis (e.g., when position 34 was mutated together with positions 11, 13, or 39), indicating that combinations involving these positions generally resulted in lower‐than‐expected affinity relative to an additive model. On the other hand, the pairing of position 18 with other positions showed both positive and negative average epistasis depending on the residue with which they were paired. Although individual mutation combinations spanned both positive and negative values, positional averaging revealed consistent trends for specific residue pairs. These patterns may reflect underlying structural or functional relationships between positions, rather than purely stochastic mutation‐specific effects.

#### 2.5.2 Affinity and epistasis in BPTI–chymotrypsin complexes

To explore how mutations interact to affect the binding affinity landscape, we analyzed double mutants by combining substitutions at one position (e.g., position 15) with mutations at other positions. By comparing the affinities of double mutants to the sum of the corresponding single‐mutant effects, we quantified epistasis and identified patterns linking changes in affinity with epistatic interactions.

**FIGURE 7:**
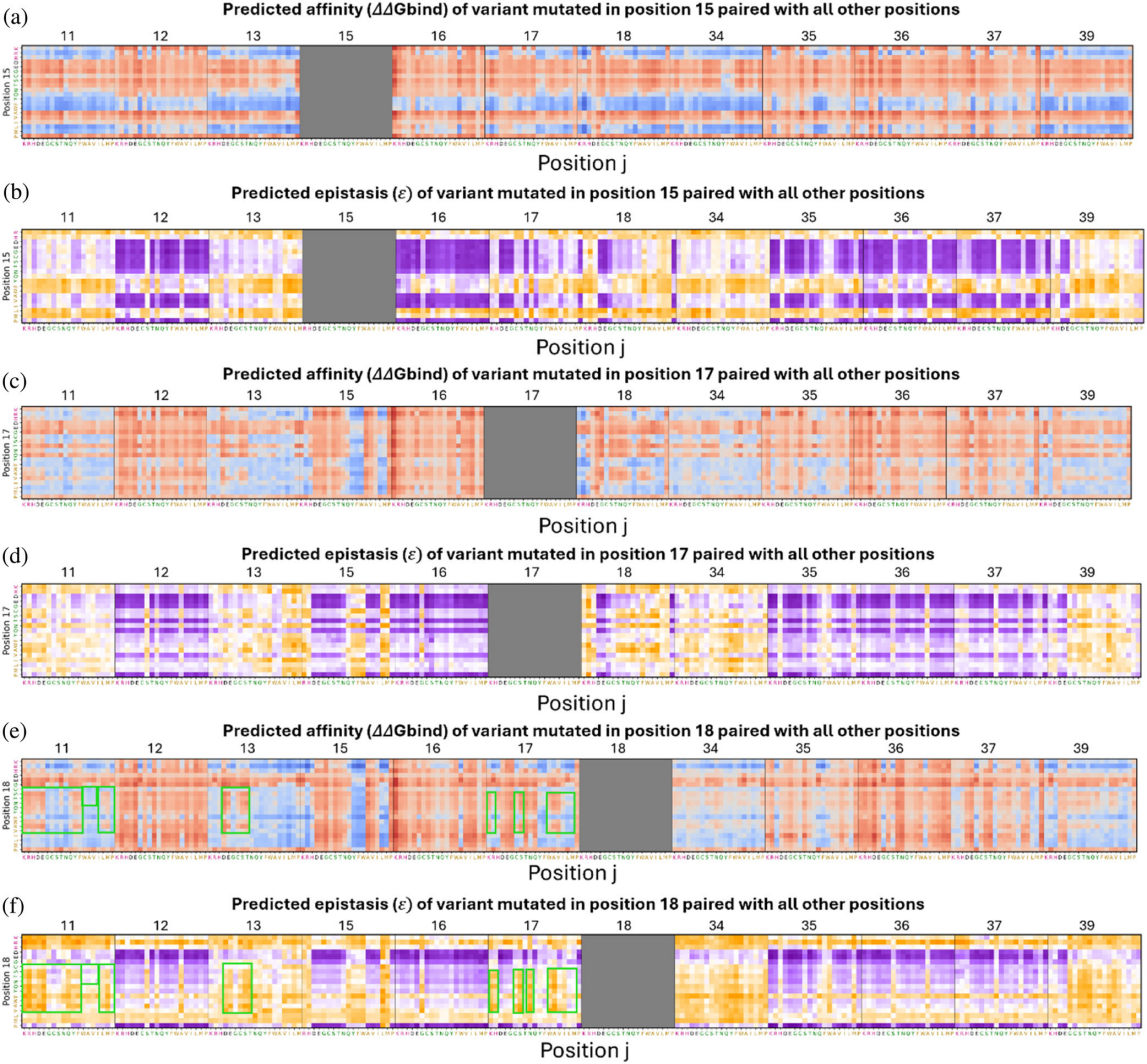
Epistasis landscape relative to the affinity landscape of double‐mutant variants at specific positions. (a), (c), (e) Affinity values (ΔΔ*G*_bind_) for variants at positions 15 (a), 17 (c), or 18 (e) with all other 11 BPTI positions. (b), (d), (f) Epistasis values (𝜀) of double mutants at positions 15 (b), 17 (d), or 18 (f) with all other 11 BPTI positions.

In some cases, affinity and epistasis were inversely correlated. For example, when Lys15 was mutated to a positively charged or an aromatic residue or to Leu or Met, affinity was enhanced but epistasis decreased. Specifically, for the variant Lys15Tyr_Arg39Ala, the values obtained for ΔΔ*G*_bind,*i*_ (Lys15Tyr) and ΔΔ*G*_bind,*j*_ (Arg39Ala) were −2.382 ± 0.265 and −1.042 ± 0.330, respectively, and that for ΔΔ*G*_bind,*ij*_ was −1.681 ± 0.350 (lower affinity than expected). If the effect of the mutations on binding had been additive, then ΔΔ*G*_bind,*ij*_ would have been the sum of ΔΔ*G*_bind,*i*_ and ΔΔ*G*_bind,*j*_ = −3.424, giving an epistasis value of 𝜀 = −1.743 ± 0.336. In contrast, negatively charged or hydrophilic residues and some hydrophobic residues at position 15 reduced affinity but increased epistasis, particularly if combined with hotspot positions (Figure 7a,b). Another example of the inverse correlation between affinity and epistasis was evident in the reduced affinity but increased epistasis upon substitution of Arg 17 with a negatively charged residue or Gly, Thr, or Pro, with the effect being stronger when the substitution was combined with a substitution in a hotspot position (Figure 7c,d).

This trend of a negative correlation between affinity and epistasis was particularly striking when a single position was mutated, as may be seen in examples of variants with two mutations of which one is in position 15. For these variants, the trend persisted across all subsequent mutations added to that position (i.e., position 15), highlighting the significant influence of the first mutation, here in position 15, on the overall epistasis and affinity outcomes of the double mutants.

We also identified cases in which affinity and epistasis were positively correlated. Specifically, as affinity increased, the epistasis of the double mutants was positive, whereas when affinity decreased, the epistasis was negative. For example, mutating Ile18 together with specific substitutions at positions 11, 13, or 17 led to reduced affinity and reduced epistasis, particularly when position 18 was occupied by hydrophilic or certain hydrophobic residues. At position 11, substitution with a charged residue or with Gln, Tyr, Phe, Leu, or Met reduced both affinity and epistasis, while Gly, Cys, and Ser residues and some hydrophobic residues tended to increase both affinity and epistasis. We observed similar trends at position 13, where substitutions with a negative residue or with Cys, Ser, or Thr reduced both affinity and epistasis. At position 17, substitutions with a positively charged residue or with Cys, Ser, Trp, Ala, Val, or Asn led to similar effects (Figure 7e,f).

The correlation between affinity and epistasis did not hold consistently across all mutation pairs introduced at the same position, indicating that the effect of a single mutation in a paired mutation was not always dominant. For some cases, the effect of one of the mutations in a pair appeared to be both context sensitive and more position‐ and mutation‐dependent than its counterpart. In these cases, the other mutation in the pair could either complement or disrupt the effect of the first mutation, leading to less predictable and more complex interactions between the mutations. We thus concluded that for mutation pairs the overall outcome of the interaction between affinity and epistasis is not dictated solely by the first mutation but is also shaped by the specific nature of the other mutation in the pair and by the interaction between the two mutations.

## 3 DISCUSSION

Mapping binding affinity landscapes in protein complexes is a challenging task, since generating and screening large mutagenesis libraries is often limited by experimental constraints. In this study, we addressed this challenge in the BPTI–chymotrypsin complex as a model system. This model was chosen for two reasons: the BPTI–chymotrypsin interaction is well characterized and the complex exhibits an intermediate binding affinity. This mid‐range affinity enables detection of both affinity‐enhancing and affinity‐weakening mutations, thereby enabling comprehensive mapping of the full binding landscape. To this end, we focused on mapping affinity landscapes in complexes of chymotrypsin with single or double BPTI mutants and on exploring the epistasis landscape for all the complexes with double BPTI mutations. We showed that the impact of mutations on binding and epistasis could be predicted using our proposed approach combining DMS, HTS, and ML techniques.

### 3.1 Factors influencing the coverage of mutational landscape

The limited/partial coverage of the theoretical double‐mutant landscape results from a combination of technical limitations in library generation and sequencing together with the biological constraints imposed by affinity‐based selection. Incomplete coverage of the 23,826 theoretical double mutants was to be expected, due to limited library representation and sequencing depth. After sorting, diversity is further reduced, since each gate contains only variants whose affinities fall within its selection range. Deeper sequencing and/or sorting a higher number of cells could recover additional, less frequent variants, yet improvements would still be constrained by sequencing costs. Although each sorted gate was sequenced to a similar depth (~200 K reads), coverage still varied substantially between gates (14%–66%), in contrast to the presorted library, which reached ~89% coverage (400 K reads). This disparity of coverage between gates suggests that reduced diversity within gates primarily reflects affinity‐based enrichment rather than insufficient sequencing depth.

### 3.2 Combining ESM2 embeddings with position‐specific encoding

Our ML models were notably quite simple (3–5 fully connected layers), but we were still able to leverage pretrained Transformer‐based ESM2 embeddings for rich contextual sequence representation. These embeddings were concatenated with position‐specific one‐hot encoding, allowing the model to integrate explicit information about substitution identity with broader sequence‐derived features. The strong predictive performance observed here suggests that this combined representation is sufficiently expressive to capture non‐linear mutational effects within a single protein system.

### 3.3 Interpreting model performance in the context of binding affinity and interface structural features

#### 3.3.1 Interpretation of model performance across affinity gates

Our ML models (for the four gates) showcased the strong predictive performance of log_2_ ER values, particularly those for the HI and LO gates, which showed high correlations with affinity values (ΔΔ*G*_bind_) for purified proteins (single‐ and double‐mutant variants) taken from the literature (Buczek et al., 2002; Castro & Anderson, 1996; Grzesiak et al., 2000; Kiczak et al., 2001; Krowarsch et al., 1999). We posit that the better correlations for the HI and LO affinity gates may be attributed to the greater change in affinity for chymotrypsin of the variants in those gates relative to the wild‐type protein. Moreover, when we examined correlations between our predictions and the affinities of purified single mutants, we observed that the test set included mostly (17 out of 27) variants mutated at position 15. Similarly, all of our evaluated double mutant variants included a mutation at position 15, and in 6 of the 10 cases the second mutation was at position 16. Position 15 is a key position in BPTI (Krowarsch et al., 1999) with a distance of less than 5 Å from some of the target residues [e.g., 4.46 Å for Met44 or 4.74 Å for Ser47 in chymotrypsin; distances were calculated using Coot 0.9.8 (Emsley et al., 2010)]. The proximity of position 15 in BPTI to the target suggests that the affinity changes (whether increasing or decreasing) caused by mutations at this position could be more pronounced compared to affinity changes resulting from mutations in other positions. This finding may explain why the HI and LO models, which capture more extreme changes, performed well for variants with mutations at position 15. It should also be noted that 10 of the 27 variants in the above‐mentioned test set were single mutations with substitutions to Ala. The known impairment of protein affinity by Ala substitutions (Meiri et al., 2024; van Petegem et al., 2008) may explain why the log_2_ ER values for the LO affinity gate give better predictions than other gates for those variants. This improved prediction ability is reflected in the experimental data showing an enrichment of variants mutated to Ala in the LO and SL populations, indicating a decrease in affinity. Combining predictions from all four affinity gates further improved the correlation of the predicted log_2_ ER values to affinity values taken from the literature, suggesting that such integration of predictions may average out gate‐specific noise and thus yield more robust predictions.

#### 3.3.2 Position‐specific hotspots and the distinct role of position 15

Mapping the affinity landscape of double‐mutant variants in BPTI‐chymotrypsin complexes revealed that mutations at positions 12, 16, and 36, and to a lesser extent at 35 and 37, consistently reduced average ΔΔ*G*_bind_ values when combined with other mutations; these positions were therefore identified as key hotspots, consistent with the findings of Heyne et al., who identified similar hotspots at positions 12, 16, 36, and 37 (Heyne et al., 2021). Although position 15 has been identified as a hotspot in BPTI complexed with other proteins, such as bovine trypsin (Heyne et al., 2020), our results showed that it does not function as a hotspot in the BPTI‐chymotrypsin complex. On the contrary, we found that mutations at position 15 may enhance the affinity of BPTI to chymotrypsin, particularly when the Lys in this position in the wild‐type BPTI is replaced with an aromatic amino acid. This differential change in affinity resulting from mutating position 15 in BPTI/chymotrypsin versus BPTI/bovine trypsin could be explained by the susceptibility to cleavage of Lys by bovine trypsin but not by chymotrypsin, which primarily recognizes aromatic amino acids, thus preventing position 15 from acting as a hotspot. This finding also highlights how mapping the full affinity landscape may enable us to better understand and draw conclusions as to how the nature (i.e., positive, negative, hydrophobic, or hydrophilic) of amino acids in the interface or at other positions may shape changes in the binding affinity in the complex.

#### 3.3.3 Position‐dependent predictability across the binding interface

Our position‐wise analysis indicated that mutational effects are not uniformly transferable across the interface. Positions 34, 39, and 13 remained relatively predictable when excluded from training, suggesting that their effects follow smoother sequence—affinity relationships that can be inferred from other residues. In contrast, hotspot positions, such as 12 and especially 16, were more difficult to predict, despite their strong enrichment in the low‐affinity gate. This reduced predictability likely reflects structurally proximal interactions that cannot be inferred solely from mutational trends at other positions. The reduced prediction performance at specific residues indicates that structural context, rather than encoding alone, underlies hotspot unpredictability. Consistent with this interpretation, we found that when variants containing mutations at the target position were included in the training set, prediction performance increased profoundly. We therefore concluded that predicting mutational effects at positions with strong structural influence on binding requires training examples that include mutations at these same positions, highlighting the limited ability of the models to extrapolate such structural effects on binding from other regions of the interface.

### 3.4 Epistasis patterns in the BPTI–chymotrypsin binding landscape

Epistasis, which is more difficult to predict than the effects of single mutations, has been explored in several PPI systems, with many studies combining DMS and predictive models (Heyne et al., 2021; Hopf et al., 2017; Judge et al., 2024; Riesselman et al., 2018), including those incorporating ML (Beck et al., 2022; Chen et al., 2023; Faure & Lehner, 2024). To the best of our knowledge, this is the first study to systematically map affinity and epistasis landscapes in the BPTI‐chymotrypsin complex by using both DMS and ML models to predict the effects of all single and double mutants, thereby providing new insights into how interactions between residues shape binding affinity.

Similar to the findings of Adams et al. (2019), who examined epistasis in an scFv antibody‐fluorescein antigen complex, and those of Judge et al. (2024), who investigated epistatic interactions within an enzyme active site, our study revealed a higher rate of positive epistasis than negative epistasis among mutation pairs, particularly when hotspot positions were paired. This finding suggests that mutations at hotspots cooperate to yield a binding affinity that is higher than what would be expected from an additive effect. Both positive and negative epistasis were most frequently observed between residues in close spatial proximity. For example, we observed epistasis between residue 36 and residue 12 or 16, which are spatially proximal. However, we also identified epistatic interactions between hotspot positions that are separated in the structure (e.g., residues 12 and 16), indicating that epistasis in our system was not restricted to neighboring sites alone. This finding is consistent with empirical reports that the magnitude of epistasis shows only a weak dependence on inter‐residue distance (Adams et al., 2019); this weak dependence can be explained by structural–energetic coupling propagated through indirect pathways, such as packing and hydrogen‐bond networks, backbone strain, or allosteric effects (Kass & Horovitz, 2002). A plausible mechanism put forward by Judge et al. (2024) is that epistasis emerges through interaction networks mediated by the binding partner, which can bridge spatially distant residues and yield substantial functional coupling, even between distant sites. The positive epistasis observed at hotspot positions of BPTI is consistent with the findings of Heyne et al. (2021), who showed that hot spot position pairing results in positive epistasis. Furthermore, the position pairs that they highlighted as negatively epistatic (i.e., 18–34 and 34–35 pairs) were likewise predicted by us, namely, the18–34 pair displayed clear negative epistasis, whereas the 34–35 pair ranged from additive to slightly negative. Notably, because our predictions provided complete coverage across substitutions, our estimates are less sensitive to missing‐variant bias than analyses based on partially sampled experimental landscapes and may therefore offer a more accurate quantification of the magnitude and consistency of negative epistasis for these pairs.

In the current study, we observed both negative and positive correlations between affinity and epistasis. In cases of negative correlation, affinity enhancement of a double mutant correlated with negative epistasis, and vice versa. For negative correlations, it seems likely that the first mutation of the pair dominates the direction of the outcome, namely, whether the affinity is enhanced or reduced, while the second mutation modulates the extent of the response, probably through allosteric adjustments that buffer the structural or energetic impact of the first mutation. We also identified cases in which affinity and epistasis were positively correlated, although this trend was not consistent across all position pairs, suggesting that the effect of a single mutation in a pair is not always dominant, similar to the concept of “sign epistasis” observed by Adams et al. (2019), where one mutation reversed the effect of another.

### 3.5 Study limitations

While our study provides valuable insights into the relationship between mutations and binding affinity, several limitations should be considered. First, the reliance on a specific experimental affinity assay introduces the potential for variability and noise in the data, as demonstrated by the discrepancies between predicted and literature‐based epistasis values. Additionally, the manual generation of gate definitions, based on our subjective judgment, and their positioning relative to the affinity range and the spread of the library may also vary, further influencing the results. Second, the fact that the data we used did not include all double‐mutation variants and was focused on specific positions, along with the limited sequencing depth of the variant library, may have restricted the scope of the predictions to single‐ and double‐mutation variants and limited our ability to predict higher‐order mutations. Expanding the training dataset by incorporating higher‐order mutations, as suggested by Meiri et al. (2024), random mutations across other positions, and deeper sequencing could improve the comprehensiveness of the data and potentially enhance model performance. Third, the limited diversity of our test set used for correlation with literature values (primarily due to the limited data available in the literature) resulted in an overrepresentation of mutations at position 15, potentially influencing the assessment of model performance. Fourth, our framework was developed and evaluated on a single protease–inhibitor complex. Because mutational effects and epistatic patterns can differ substantially across interfaces, conformational regimes, and experimental contexts, the extents to which the observed performance and derived conclusions generalize to other protease–inhibitor systems have not yet been tested. Systematic benchmarking on additional protease–inhibitor pairs, ideally spanning diverse interface architectures and affinity ranges, will be required to assess transferability. Fifth, epistasis was not modeled as a direct output or calibrated directly against experimental epistasis measurements; instead, it was derived from model‐predicted ΔΔGbind values. An epistasis‐specific calibration (analogous to our ΔΔ*G*_bind_ calibration) is currently not feasible, because the available experimental epistasis measurements are insufficient. Therefore, we cannot exclude the possibility that some predicted epistatic patterns are partially influenced by smoothing/interpolation effects of the inferred ΔΔ*G*_bind_ surface, and broader experimental validation will be required. Lastly, our models are limited by their positional generalizability. The one‐hot encoding represents positions independently, restricting explicit modeling of structural relationships between residues, and averaging ESM embeddings across positions may attenuate single‐residue perturbations and obscure the structural origin of mutational effects. These constraints become particularly evident under strict position‐wise exclusion, where one‐hot columns for a given position are never observed during training. Although prior work (Gurusinghe et al., 2024) has incorporated structural ESM‐IF1 models based solely on the wild‐type structure, we reasoned that such representations may be more informative for comparisons across various complexes than for one complex. We therefore focused exclusively on sequence‐derived features. In the future, incorporation of variant‐specific structural representations or position‐aware pooling strategies may further improve prediction.

### 3.6 Conclusions and future perspectives

In summary, our work provides valuable insights into the relationship between mutations and binding affinity, advancing our understanding of PPIs and affinity landscapes. By mapping epistasis and the affinity of single and double mutants for chymotrypsin, we contribute to the growing body of knowledge on how specific mutations influence PPIs. Importantly, we establish a foundation for expanding binding landscape modeling to higher‐order mutations. Generalization beyond the current protein system will require systematic validation across additional protease–inhibitor pairs.

## 4 METHODS

### 4.1 Computational analysis of HTS data

We translated the DNA sequences of the pre‐sorted library and the four sorted library fractions into the amino‐acid sequences that they encode. Thereafter, we filtered out sequences that lacked the TAGC primer (the start of the protein‐coding region) and also sequences that were too short (less than 162 bp corresponding to 54 amino acids), contained unrecognized DNA base pairs, or carried mutations outside the 12 predefined positions (at the protein level); the BPTI sequence of amino acids 1–54 was used as a reference (Kuroda & Kim, 2000).

Following amino‐acid translation and sequence filtering, we counted the number of occurrences of each variant in each library fraction. Then, we calculated the frequency of each variant by dividing the count by the total number of occurrences of the variant in that fraction:

(1) $$f_{\text{mutj},\text{gate}\;A}=\frac{\#\text{r}\text{eads}\;\mathrm{mut}\;j\;\text{in gate}\;A}{\sum_{j=1}^{n}\#\text{reads in gate}\;A}$$

where “#readsjin gate” is the number of reads of a variant *j* in gate *A*, and “$\sum_{j=1}^{n}\#r\text{eads in gate}\;A$” is the sum of all filtered reads for all variants in the same gate.

Next, to compare the frequencies of each variant to that of the wild type in the same library fraction, we calculated normalized frequency (NF) of each variant in each library fraction:

(2) $$\;{\mathrm{NF}}_{\mathrm{mut}\;j,\text{gate}\;A}=\frac{f_{\mathrm{mut}\;j,\;\text{gate}\;A}}{f_{\mathrm{wt},\;\text{gate}\;A}}$$

which is the ratio of the frequency of a given variant in gate *A* to the frequency of wild‐type BPTI in the same gate.

Then, based on the NFs, we calculated the ERs of each variant in the BPTI library as follows:

(3) $$\;{\mathrm{ER}}_{\mathrm{mut}\;j,\text{gate}\;A}=\frac{{\mathrm{NF}}_{\mathrm{mut}\;j,\;\text{gate}\;A}}{{\mathrm{NF}}_{\mathrm{mut}\;j,\;\mathrm{pre}-\text{sort}}}$$

where ER was defined as the ratio between the mutant's NF in a specific gate and its frequency in the pre‐sorted library.

### 4.2 ML model architecture, input representation, and hyperparameter search

We explored multiple input representations and model architectures for predicting log_2_ ER values for each affinity gate (HI, WT, SL, and LO). Input representations included: (i) one‐hot encoding of the 12 mutated interface positions (20 × 12 = 240 features), (ii) pretrained ESM2 embeddings extracted from the full‐length protein sequence, and (iii) combinations of one‐hot and ESM‐derived features. For ESM‐based representations, we evaluated residue‐level embeddings of 640 dimensions. We explored several aggregation strategies across the 12 mutated positions, including averaging (for 480 and 640 parameters), combined average–max–min pooling, and non‐aggregated positional embeddings. In combined models, we concatenated the embedding‐derived features with flattened one‐hot encoding.

We evaluated multiple ML models, including ridge regression, support vector regression (linear and polynomial kernels), random forest, convolutional neural networks, recurrent neural networks, and fully connected neural networks (NN). For neural network‐based models, architectures ranged from 3 to 5 fully connected layers with ReLU activation and a linear output neuron.

**FIGURE 8:**
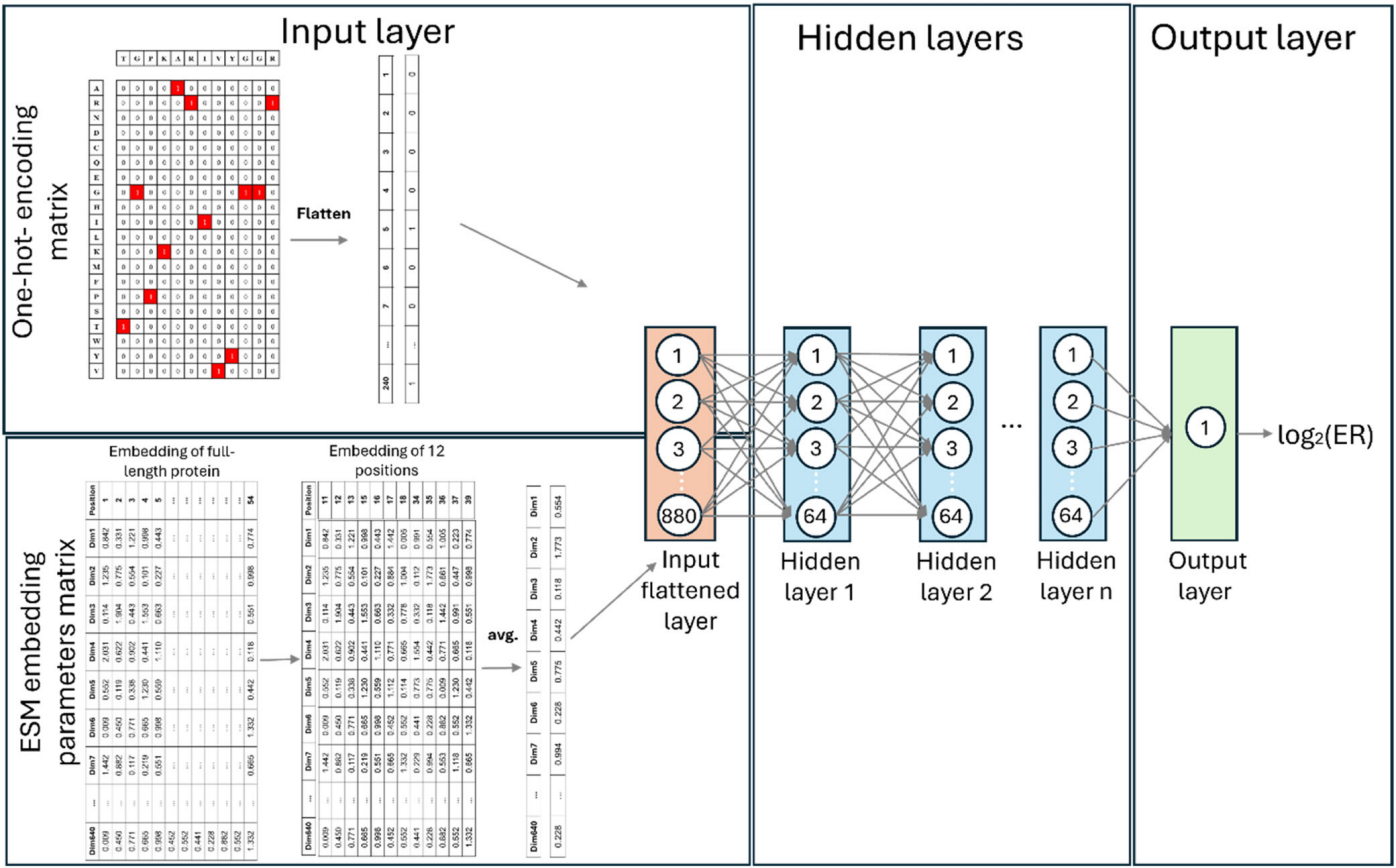
Architecture of our models to predict log_2_ ER. The input is a one‐hot encoded matrix of the 12 mutated binding residues concatenated to ESM base 640 embedding parameters vector after average embedding over the 12 positions. The model consists of 3–5 fully connected layers depending on the specific gate, with a ReLU activation function. The output layer is a single neuron with linear activation.

For model hyperparameter tuning, we randomly sampled multiple hyperparameter configurations from a predefined search space, where the number of evaluated configurations scaled according to the model's complexity and computational cost (Table S3). Hyperparameter selection was based on the Pearson correlation between predicted and experimental log_2_ ER values on the validation set. The dataset was partitioned based on sequencing depth to ensure high‐confidence evaluation. Variants were ranked according to the total read count (pre‐sorted library + specific gate), and the top 10% were designated as the test set, with the subsequent 10% being used as the validation set. The remaining 80% constituted the training set. This strategy ensured that model evaluation and hyperparameter selection were performed on variants with the most reliable enrichment measurements. To enhance robustness, each model was trained using a random‐ensemble initialization strategy (10 independent initializations), and predictions were averaged. We selected the final model architecture and input representation based on average performance over the validation sets of the gates. The selected input representation and architecture was a one‐hot encoding matrix concatenated to averaged ESM 640 embedding parameters of the 12 positions following a fully connected NN (Figure 8 and Table S3). Following architecture, input representation, and hyperparameter selection, the selected model was re‐trained on 90% of the data (training + validation sets) and evaluated on the held‐out 10% test set.

### 4.3 Model evaluation under alternative data partitioning schemes

After selecting the final model based on the read‐count‐based split, we further evaluated its robustness under alternative partitioning strategies. These included position‐wise holdout, in which all variants containing a mutation at a given position were excluded from training and used exclusively for testing. In addition, we performed random partitioning in which 10% of the dataset was held out as an independent test set. In these analyses, the finalized architecture and hyperparameters were kept fixed, and no further tuning was performed.

### 4.4 Converting HTS‐based ER values to absolute affinities

To obtain an equation for converting ER values from the four sorted populations into ΔΔ*G*_bind_ values, we used 27 available experimental ΔΔ*G*_bind_ measurements of single‐mutant variants that we found in the literature (Castro & Anderson, 1996; Krowarsch et al., 1999) for the BPTI–chymotrypsin complex. We used a linear equation with four variables, which are the log_2_ ER values predicted by our ML models (Equation (4)). Their coefficients and a free bias term were learned by minimizing the mean squared error over the experimental ΔΔ*G*_bind_ measurements. We used the obtained equation to calculate ΔΔ*G*_bind_ values for all the single and double BPTI mutants, as follows:

(4) $$\Delta \Delta G_{\text{bind}}=\begin{array}{l} –0.397\;\log_{2}\;{\mathrm{ER}}_{\mathrm{HI}}–0.348\;\log_{2}\;{\mathrm{ER}}_{\mathrm{SL}}\\ +\;0.184\;\log_{2}\;{\mathrm{ER}}_{\mathrm{WT}}+0.597\;\log_{2}\;{\mathrm{ER}}_{\mathrm{LO}}–0.560 \end{array}$$

### 4.5 Experimental validation of ΔΔ*G*_bind_ predictions

To experimentally validate the ΔΔ*G*_bind_ predictions, we used variants with experimentally measured ΔΔ*G*_bind_ values as an independent test set on which the models had not been trained. Each test‐set variant contained a single or double mutation in one or two of the 12 residue positions mutated in our library (i.e., positions 11–13, 15–18, 34–36, 39). We calculated the correlation between the ΔΔ*G*_bind_ values and the log_2_ ER predictions for each gate or the combined ΔΔ*G*_bind_.

### 4.6 Additive‐baseline comparison for double‐mutant ΔΔ*G*_bind_ prediction

To assess whether direct double‐mutant ΔΔ*G*_bind_ predictions improved upon a simple additive approximation, we compared them with an additive baseline using the 10 double‐mutant variants with experimentally measured ΔΔ*G*_bind_ values. To ensure an independent evaluation, we excluded these double mutants and their corresponding single‐mutant variants from the training set. For each double mutant, the additive baseline was calculated as the sum of the predicted ΔΔ*G*_bind_ values of the two corresponding single mutants. The direct prediction was defined as the predicted ΔΔ*G*_bind_ value of the double‐mutant sequence obtained from the combined affinity‐gate model. We compared both the direct and additive predictions with the experimental ΔΔ*G*_bind_ values using Pearson correlation. To account for the limited size of the validation set, we performed an exhaustive subsampling analysis in which Pearson correlation was calculated for all possible subsets of 8, 9, and 10 double mutants, sampled without replacement. We report the mean ± SD across all tested subsets.

### 4.7 Analysis of affinity and cooperativity

Affinity predictions were calculated using an ensemble‐based framework in which each ensemble member was processed independently throughout the entire prediction pipeline. For each seed, the predicted ERs from the four selection gates (HI, WT, SL, and LO) were combined using the fitted linear model equation (Equation (4)) to generate a seed‐specific ΔΔ*G*_bind_ estimate. These ΔΔ*G*_bind_ values were then normalized relative to the WT sequence by subtracting the predicted WT value from the predicted value of each variant, such that the WT was assigned a value of 0 for each seed. Epistasis was subsequently calculated separately for each ensemble member. Specifically, the epistasis value, denoted by 𝜀*ij*, was defined as the difference between the sum of the ΔΔ*G*_bind_ values of the corresponding single mutants and the ΔΔ*G*_bind_ value of the double mutant, as shown in Equation (5).

(5) $$\text{𝜀}\mathrm{ij}=\Delta \Delta G_{\text{bind},i}+\Delta \Delta G_{\text{bind},j}–\Delta \Delta G\text{bind},\mathrm{ij}$$

The final reported ΔΔ*G*_bind_ and epistasis (𝜀*ij*) values represent the mean across ensemble members, and the corresponding standard deviations across seeds were used as empirical estimates of predictive uncertainty.

### 4.8 Modeling and stability calculations

We modeled the mutations and calculated the relative stability (ΔΔ*G*_bind_) of every point mutation relative to the WT with FoldX version 5.1 (Delgado et al., 2019; Schymkowitz et al., 2005). As a model of the WT, we used the crystal structure of the complex between bovine alpha‐chymotrypsin and BPTI (PPDB ID 1MTN; Capasso et al., 1997). A negative ΔΔ*G* value indicated that the mutant was more stable than the WT, while a positive ΔΔ*G* value suggested that the mutant was less stable than the WT. PyMOL Molecular Graphics System, Version 3.1.6.1 (Schrödinger, LLC.) was used for visual analysis of selected mutations and for preparation of the structural figures.

## AUTHOR CONTRIBUTIONS

**Yaron Orenstein:** Conceptualization; methodology; formal analysis; supervision; writing – original draft; writing – review and editing; funding acquisition. **Noam Tzuri:** Conceptualization; methodology; investigation; software; data curation; formal analysis; writing – original draft; writing – review and editing; validation. **Niv Papo:** Conceptualization; methodology; formal analysis; funding acquisition; writing – original draft; writing – review and editing; project administration; resources; visualization; supervision. **Itamar Kass:** Formal analysis; writing – original draft; writing – review and editing.

## CONFLICT OF INTEREST STATEMENT

The authors declare no conflict of interest.

## Supporting information


**Table S1:** Prediction performance of our gate‐specific ML models over various architecture and input representation combinations. For each gate, we trained each model on the 80% least‐frequent variants. We used the 10% most frequent variants as the test set. We used the second‐most frequent 10% as the validation set for hyperparameter and model optimization.
**Figure S1:** Model performance assessed by correlation with experimental binding affinities tested on a random dataset. We randomly partitioned each dataset into 90% training and 10% test sets. We trained each model on the training set and evaluated it on the test set of the (A) HI, (B) WT, (C) SL, and (D) LO gates. *N*—number of datapoints in the test set, *R*—Pearson correlation, RMSE—root mean square error.
**Figure S2:** Model performance assessed by correlation with experimental binding affinities. Pearson correlations between ML‐based log_2_ ER predictions and experimentally measured ΔΔ*G*_bind_ values of purified proteins for 27 single‐mutation variants. (A) HI, (B) WT, (C) SL, and (D) LO gates. *N*—number of datapoints in the test set, *R*—Pearson correlation.
**Figure S3:** Model performance assessed by correlation with experimental binding. Pearson correlations between ML‐based log_2_ ER predictions and experimentally measured ΔΔ*G*_bind_ values of purified proteins for 10 double‐mutation variants. (A) HI, (B) WT, (C) SL, and (D) LO gates. *N*—number of datapoints in the test set, *R*—Pearson correlation.
**Figure S4:** Pearson correlations for combined gates. ΔΔ*G*_bind_ based on ML‐predicted log_2_ ER (i.e., predicted ΔΔ*G*_bind_) and experimentally measured ΔΔ*G*_bind_ based on purified proteins of (A) 27 single mutation variants or (B) 10 double‐mutation variants. *N*—number of datapoints in the test set, *R*—Pearson correlation, RMSE—root mean square error.
**Figure S5:** Structural details of models of the chymotrypsin: BPTI complex. Chymotrypsin (green) and BPTI (cyan) are rendered as cartoons. Position 15 of BPTI is rendered as sticks, and close side chains are rendered as lines (with oxygen in red and nitrogen in blue). Hydrogen bonds between position 15 and surrounding residues are depicted as dashed yellow lines.
**Table S2:** Prediction performance of the gate‐specific ML models for each position. We defined all variants containing mutations at specific positions as the test set and trained the models on the remaining data.
**Figure S6:** Effect of including target‐position variants in the training set on model performance. The test sets were composed of the 10% most frequent variants stratified by mutated positions. We compared two training schemes: one excluding from the training set all variants with mutations at the target position (orange), and one including all non‐test variants with mutations at the target position in the training set (blue). The plotted values represent Pearson correlations between predicted and measured log_2_ ER values, averaged across four affinity gates.
**Table S3:** Hyperparameter search space for different architectures.

## Data Availability

HTS data used in this study are publicly available in NCBI Gene Expression Omnibus (GEO) repository under accession number GSE325790: https://www.ncbi.nlm.nih.gov/geo/query/acc.cgi?acc=GSE325790. The code trained models and processed datasets are available in GitHub at https://github.com/OrensteinLab/BPTI-epistasis. The protein structures used in this study are publicly available from the Protein Data Bank (PDB). The following accession codes correspond to the structural models analyzed in this manuscript: 1MTN.
