# Supplementary material for: Machine‐learning prediction of affinity and epistasis in the bovine pancreatic trypsin inhibitor–chymotrypsin complex
Source: Protein Sci. 2026 Jun 5;35(7):e70660. doi: 10.1002/pro.70660 (PMC13238754; doi:10.1002/pro.70660)
Supplement: Supplementary file 1 — Table S1: Prediction performance of our gate‐specific ML models over various architecture and input representation combinations. For each gate, we trained each model on the 80% least‐frequent variants. We used the 10% most frequent variants as the test set. We used the second‐most frequent 10% as the validation set for hyperparameter and model optimization. Figure S1: Model performance assessed by correlation with experimental binding affinities tested on a random dataset. We randomly partitioned each dataset into 90% training and 10% test sets. We trained each model on the training set and evaluated it on the test set of the (A) HI, (B) WT, (C) SL, and (D) LO gates. N—number of datapoints in the test set, R—Pearson correlation, RMSE—root mean square error. Figure S2: Model performance assessed by correlation with experimental binding affinities. Pearson correlations between ML‐based log2 ER predictions and experimentally measured ΔΔG bind values of purified proteins for 27 single‐mutation variants. (A) HI, (B) WT, (C) SL, and (D) LO gates. N—number of datapoints in the test set, R—Pearson correlation. Figure S3: Model performance assessed by correlation with experimental binding. Pearson correlations between ML‐based log2 ER predictions and experimentally measured ΔΔG bind values of purified proteins for 10 double‐mutation variants. (A) HI, (B) WT, (C) SL, and (D) LO gates. N—number of datapoints in the test set, R—Pearson correlation. Figure S4: Pearson correlations for combined gates. ΔΔG bind based on ML‐predicted log2 ER (i.e., predicted ΔΔG bind) and experimentally measured ΔΔG bind based on purified proteins of (A) 27 single mutation variants or (B) 10 double‐mutation variants. N—number of datapoints in the test set, R—Pearson correlation, RMSE—root mean square error. Figure S5: Structural details of models of the chymotrypsin: BPTI complex. Chymotrypsin (green) and BPTI (cyan) are rendered as cartoons. Position 15 of BPTI is rendered as sticks, and close sid [file PRO-35-e70660-s001.docx]

**Supplementary information**

**Machine-Learning Prediction of Affinity and Epistasis in the Bovine Pancreatic Trypsin Inhibitor–Chymotrypsin Complex**

Noam Tzuri, Itamar Kass, Yaron Orenstein, and Niv Papo

**Table S1:** **Prediction performance of our gate-specific ML models over various architecture and input representation combinations.** For each gate, we trained each model on the 80% least-frequent variants. We used the 10% most frequent variants as the test set. We used the second-most frequent 10% as the validation set for hyperparameter and model optimization.

| **Avg Pearson** | **HI** | **WT** | **SL** | **LO** | **Model** |
| --- | --- | --- | --- | --- | --- |
|  | **Pearson correlation on validation set** | | | |  |
| 0.822 | 0.864 | 0.824 | 0.837 | 0.761 | One-hot+ NN |
| 0.741 | 0.782229 | 0.789211 | 0.758045 | 0.633048 | One-hot+ random forest |
| 0.714 | 0.782 | 0.789 | 0.758 | 0.633 | One-hot + SVR linear kernel |
| 0.755 | 0.810 | 0.743 | 0.726 | 0.577 | One-hot+ SVR poly kernel |
| 0.822 | 0.816 | 0.783 | 0.769 | 0.653 | One-hot+ CNN+ NN |
| 0.785 | 0.860 | 0.826 | 0.835 | 0.769 | One-hot+ RNN |
| 0.800 | 0.799 | 0.769 | 0.816 | 0.757 | ESM avg. 480 +NN* |
| 0.815 | 0.838 | 0.789 | 0.809 | 0.765 | ESM avg. 480 +max+min +NN* |
| 0.812 | 0.851 | 0.819 | 0.819 | 0.771 | ESM avg.640 +NN* |
| 0.827 | 0.855 | 0.806 | 0.817 | 0.768 | ESM no avg.640 +NN* |
| 0.715 | 0.866 | 0.823 | 0.841 | 0.777 | One-hot+ ridge regression |
| 0.826 | 0.809 | 0.747 | 0.728 | 0.577 | One-hot+ ESM avg. 640 +NN* |
| *480/640 refer to the embedding dimension in the ESM2. | | | | | |

**
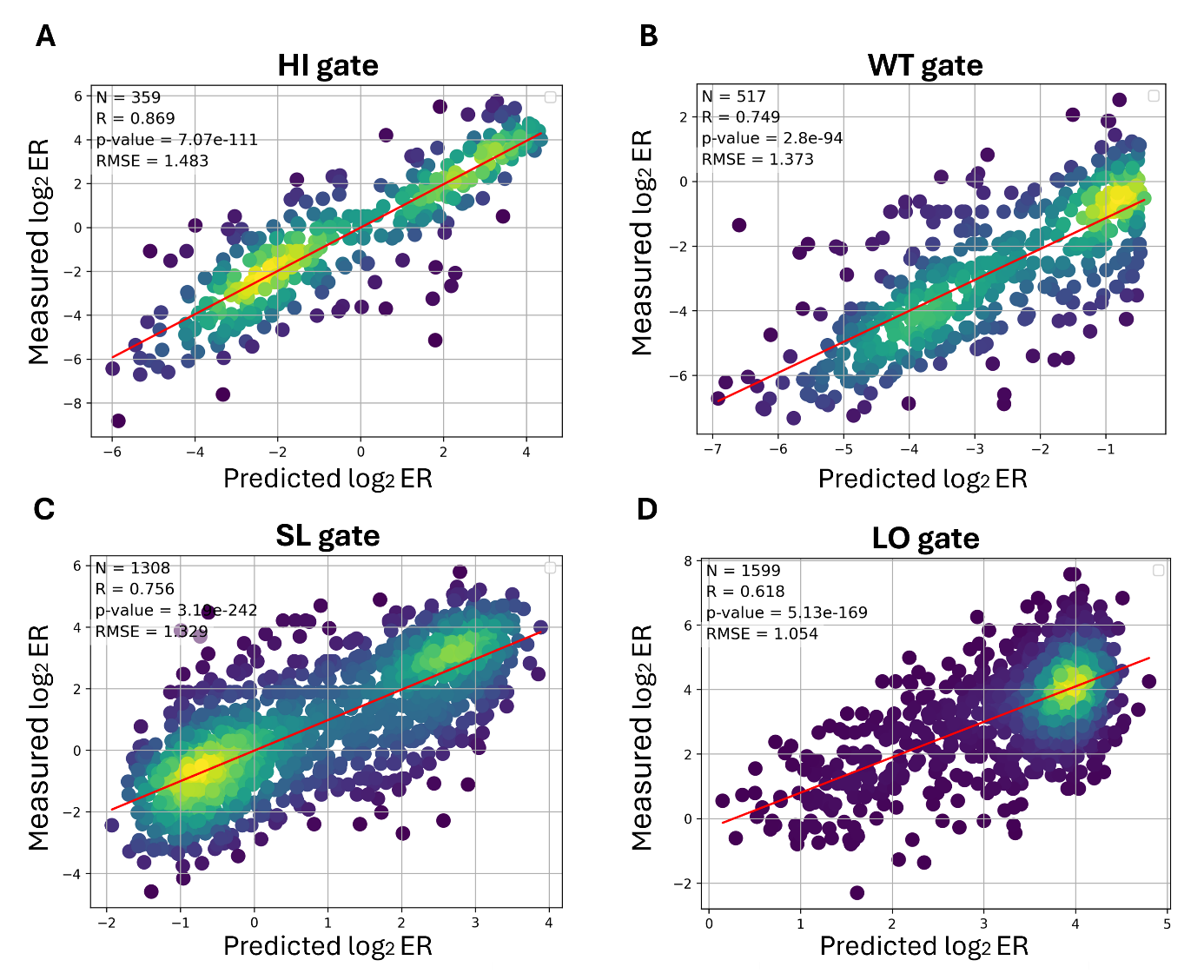
**

**Figure S1:** **Model performance assessed by correlation with experimental binding affinities** **tested on a random dataset.** We randomly partitioned each dataset into 90% training and 10% test sets. We trained each model on the training set and evaluated it on the test set of the (A) HI, (B) WT, (C) SL, and (D) LO gates. N - number of datapoints in the test set, R - Pearson correlation, RMSE - root mean square error.

**
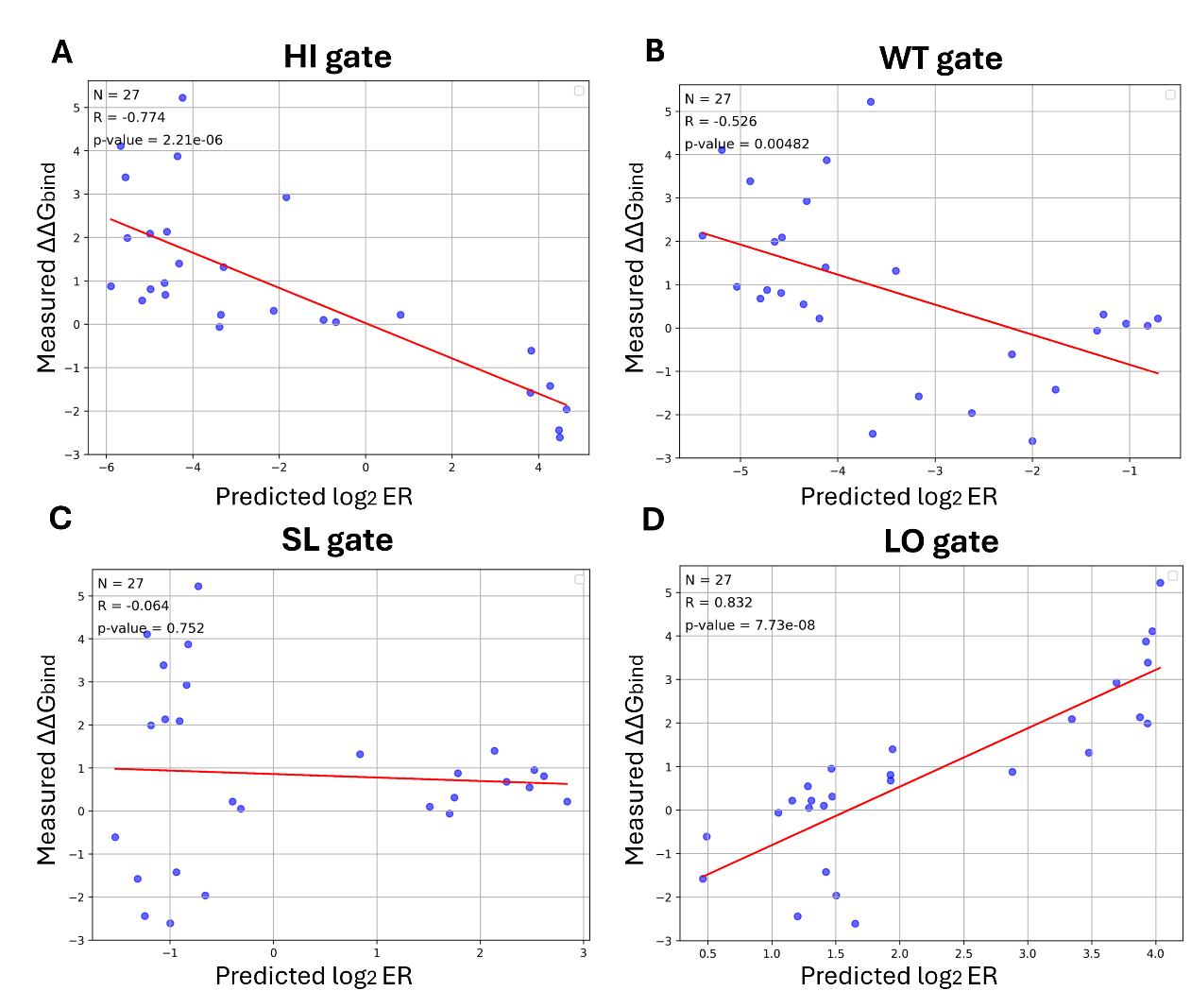
**

**Figure S2:** **Model performance assessed by correlation with experimental binding affinities**. Pearson correlations between ML-based log_2_ ER predictions and experimentally measured ΔΔG_bind_ values of purified proteins for 27 single-mutation variants. (A) HI, (B) WT, (C) SL, and (D) LO gates. N - number of datapoints in the test set, R - Pearson correlation.


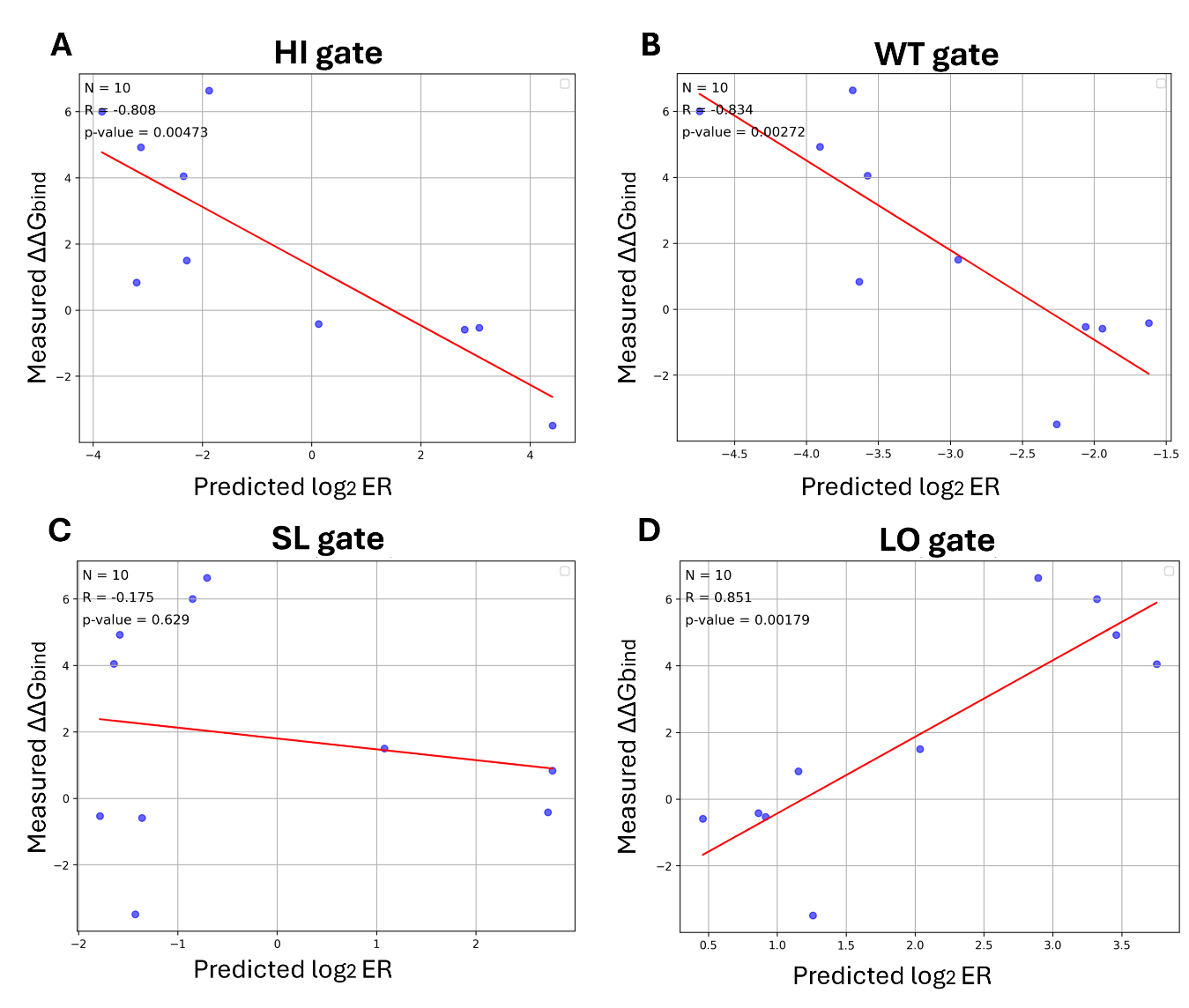


**Figure S3: Model performance assessed by correlation with experimental binding**. Pearson correlations between ML-based log_2_ ER predictions and experimentally measured ΔΔG_bind_ values of purified proteins for 10 double-mutation variants. (A) HI, (B) WT, (C) SL, and (D) LO gates. N - number of datapoints in the test set, R - Pearson correlation.


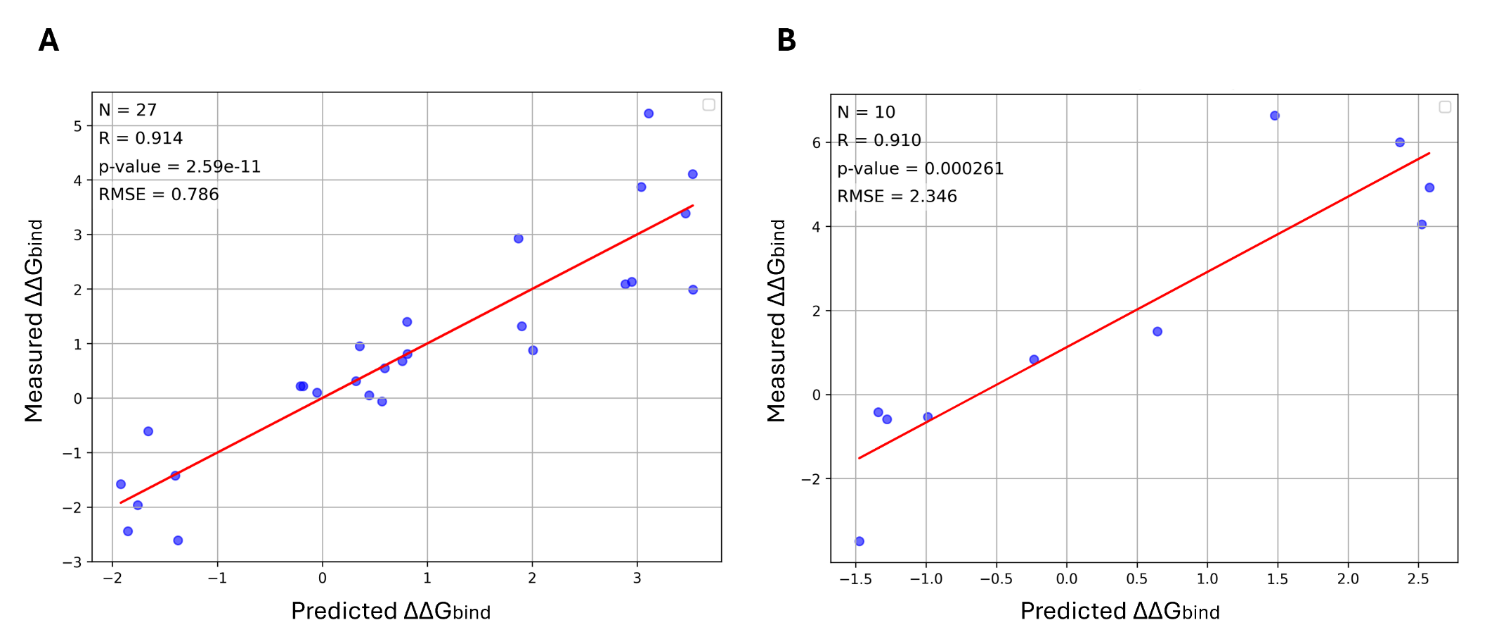


**Figure S4:** **Pearson correlations for combined gates.** ΔΔG_bind_ based on ML-predicted log_2_ ER (i.e., predicted ΔΔG_bind_) and experimentally measured ΔΔG_bind_ based on purified proteins of (A) 27 single mutation variants or (B) 10 double-mutation variants. N - number of datapoints in the test set, R - Pearson correlation, RMSE - root mean square error.

| 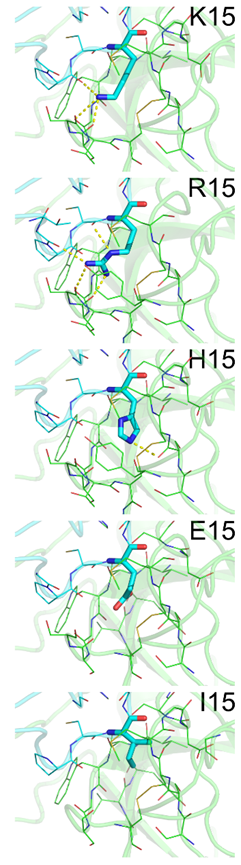 | **Figure S5: Structural details of models of the chymotrypsin:BPTI complex.** Chymotrypsin (green) and BPTI (cyan) are rendered as cartoons. Position 15 of BPTI is rendered as sticks, and close side chains are rendered as lines (with oxygen in red and nitrogen in blue). Hydrogen bonds between position 15 and surrounding residues are depicted as dashed yellow lines. |
| --- | --- |

**Table S2:** **Prediction performance of the gate-specific ML models for each position.** We defined all variants containing mutations at specific positions as the test set and trained the models on the remaining data.

| **avg. RMSE** | **avg. R** | **LO** | | | **SL** | | | **WT** | | | **HI** | | |  | |
| --- | --- | --- | --- | --- | --- | --- | --- | --- | --- | --- | --- | --- | --- | --- | --- |
|  |  | **RMSE** | **R** | **Test size** | **RMSE** | **R** | **Test size** | **RMSE** | **R** | **Test size** | **RMSE** | **R** | **Test size** | **Posi­tion** |  |
| 1.996 | 0.551 | 1.422 | 0.534 | 2096 | 1.896 | 0.494 | 2431 | 1.899 | 0.588 | 1225 | 2.767 | 0.587 | 866 | 11 |  |
| 1.856 | 0.341 | 1.544 | 0.245 | 3102 | 1.906 | 0.242 | 1917 | 1.948 | 0.364 | 611 | 2.025 | 0.511 | 270 | 12 |  |
| 1.764 | 0.602 | 1.266 | 0.571 | 2280 | 1.686 | 0.570 | 2450 | 1.709 | 0.582 | 1271 | 2.393 | 0.686 | 960 | 13 |  |
| 2.541 | 0.349 | 1.691 | 0.252 | 2519 | 1.904 | 0.293 | 1877 | 2.431 | 0.272 | 884 | 4.137 | 0.577 | 951 | 15 |  |
| 2.316 | 0.175 | 1.779 | 0.170 | 2859 | 1.840 | 0.112 | 1419 | 2.366 | 0.146 | 334 | 3.280 | 0.272 | 149 | 16 |  |
| 1.872 | 0.520 | 1.379 | 0.465 | 2591 | 1.840 | 0.444 | 2337 | 1.835 | 0.569 | 828 | 2.435 | 0.601 | 428 | 17 |  |
| 2.353 | 0.520 | 1.414 | 0.497 | 2413 | 1.819 | 0.529 | 2331 | 2.383 | 0.490 | 825 | 3.794 | 0.562 | 673 | 18 |  |
| 1.677 | 0.654 | 1.170 | 0.622 | 2196 | 1.663 | 0.575 | 2410 | 1.936 | 0.617 | 1254 | 1.939 | 0.800 | 930 | 34 |  |
| 1.704 | 0.562 | 1.238 | 0.366 | 2807 | 1.814 | 0.453 | 2071 | 1.512 | 0.689 | 558 | 2.253 | 0.740 | 270 | 35 |  |
| 1.510 | 0.485 | 1.179 | 0.314 | 3270 | 1.522 | 0.458 | 1893 | 1.722 | 0.489 | 536 | 1.617 | 0.680 | 281 | 36 |  |
| 1.870 | 0.378 | 1.433 | 0.335 | 3141 | 1.808 | 0.353 | 2263 | 1.670 | 0.442 | 631 | 2.569 | 0.382 | 339 | 37 |  |
| 1.709 | 0.628 | 1.199 | 0.606 | 2490 | 1.696 | 0.547 | 2635 | 1.740 | 0.626 | 1156 | 2.202 | 0.732 | 862 | 39 |  |

R - Pearson correlation, RMSE- [root mean square error](https://www.google.com/search?q=Root+Mean+Square+Error&oq=rmse+&gs_lcrp=EgZjaHJvbWUyBggAEEUYOTIHCAEQABiABDIHCAIQABiABDIHCAMQABiABDIHCAQQABiABDIHCAUQABiABDIHCAYQABiABDIHCAcQABiABDIHCAgQABiABDIHCAkQABiABNIBCDMxMThqMGo3qAIAsAIA&sourceid=chrome&ie=UTF-8&mstk=AUtExfADm0HU5ohAHkdLCbrWn6QTuh83oukJi2h3fqhUBAJyx8EpJHWw4219ltTn5MkLdBX7XiBhqGwOvGJv3zVl_funiefP2U6DK0WDx45ZUdlielAoL7Jh6cR2IoopqLBYDxRroLHjJo6Z5oLIBYTjzd5yxEzeIqBen0J0WkPL5yjdM38&csui=3&ved=2ahUKEwikksvFrfKSAxVshv0HHSrwDUYQgK4QegQIARAE).


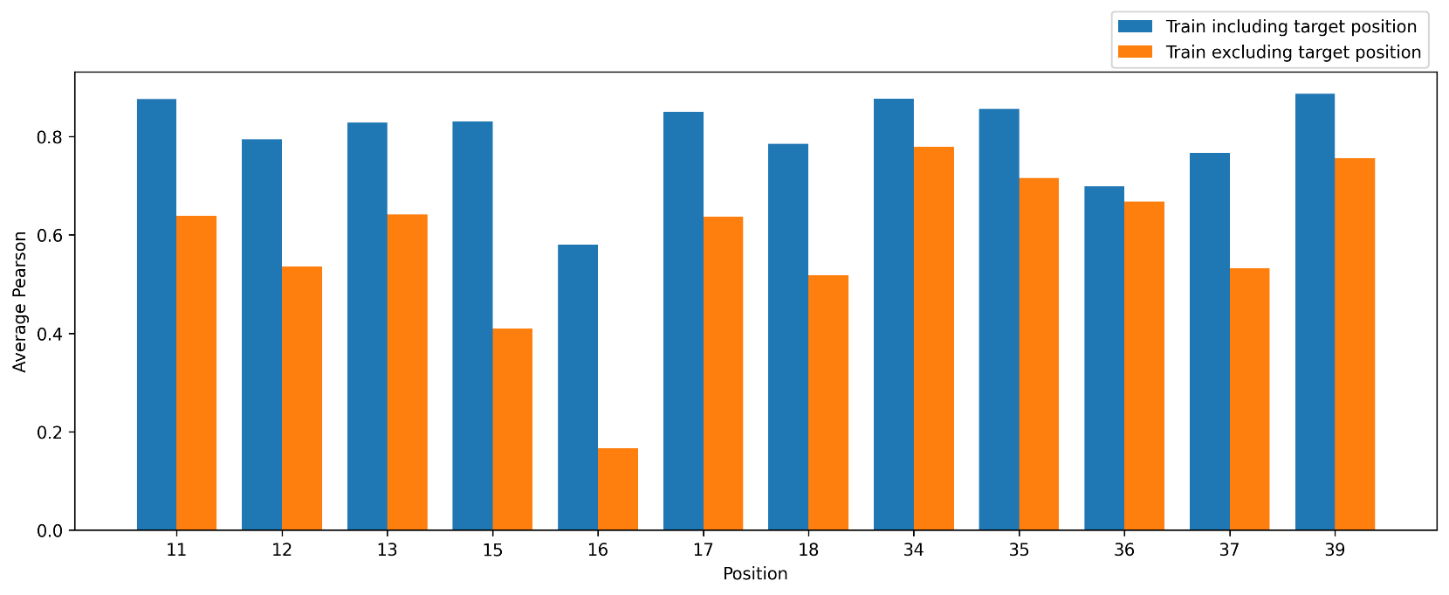


**Figure S6: Effect of including target-position variants in the training set on model performance**. The test sets were composed of the 10% most frequent variants stratified by mutated positions. We compared two training schemes: one excluding from the training set all variants with mutations at the target position (orange), and one including all non-test variants with mutations at the target position in the training set (blue). The plotted values represent Pearson correlations between predicted and measured log_2_ ER values, averaged across four affinity gates.

**Table S3:** **Hyperparameter search space for different architectures.**

| Hyperparameter for fully connected NN | Search range | Hyperparameter for linear kernel SVR | Search range |
| --- | --- | --- | --- |
| Batch size | {32, 64, 128, 256, 512} | C | {0.1, 1, 10, 50, 100} |
| Number of epochs | {10, 20, 30, 40, 50} | Epsilon | {0.01, 0.1, 0.5, 1.0, 2.0} |
| Optimizer learning rate | {10^-5^, 10^-4^, 5×10^-4^, 10^-3^} | Hyperparameter for RNN | Search range |
| Layers in the architecture | {3, 4, 5} | Rnn_type | {GRU, LSTM} |
| Neurons in the architecture's layers | {32, 64, 128, 256} | Rnn_layers | {1, 2} |
| Dropout | {0,0.1, 0.2, 0.3, 0.4, 0.5} | Units | {32, 64, 128} |
| Hyperparameter for ridge regression | Search range | Bidirectional | {False, True} |
| Alpha | {10^-3^, 10^-2^, 0.1, 1, 10, 100, 1000} | Recurrent_dropout | {0.0, 0.1} |
| Hyperparameter for poly kernel SVR | Search range | Dropout_rate | {0.1, 0.2, 0.35} |
| C | {0.1, 1, 10} | Dense_layers | {0, 1, 2} |
| Epsilon | {0.01, 0.1, 0.5} | Dense_units | {64, 128, 256} |
| Degree | {2, 3, 4} | Learning_rate | {10^-5^, 10^-4^, 5×10^-4^, 10^-3^} |
| Coef0 | {0, 0.5, 1} | Batch_size | {32, 64, 128} |
| Hyperparameter for CNN | Search range | Hyperparameter for random forest | Search range |
| Arch | {"conv_layers": 1, "filters": (32,)}, {"conv_layers": 1, "filters": (64,)}, {"conv_layers": 2, "filters": (32, 64)},  {"conv_layers": 2, "filters": (64, 128)}, {"conv_layers": 3, "filters": (32, 64, 128)} | N_estimators | {300, 600, 1000} |
| Kernel_size | {2, 3} | Max_depth | {None, 5, 10, 20, 40} |
| Dense_layers | {1, 2} | Min_samples_split | {2, 5, 10} |
| Dense_units | {64, 128, 256} | Max_features | {"sqrt", "log2", 0.3, 0.5, 1.0} |
| Dropout_rate | {0.2, 0.35, 0.5} |  | |
| Learning_rate | {10^-5^, 10^-4^, 5×10^-4^, 10^-3^} |  |  |
| Batch_size | {32, 64, 128} |  |  |
